# Development and external validation of a nomogram for predicting the risk of developing esophageal cancer based on a questionnaire: a multicenter case-control study

**DOI:** 10.3389/fonc.2025.1684561

**Published:** 2025-12-04

**Authors:** Yaling Zhang, Tao Sun, Xiaoqi Chen, Dongdong Li, Feifan Feng, Lihan Zhang, Xiaoyu Tang, Jianping Liu, Yuling Zheng

**Affiliations:** 1 Department of Oncology, The First Affiliated Hospital of Henan University of Chinese Medicine, Zhengzhou, China; 2 Collaborative Innovation Center of Prevention and Treatment of Major Diseases by Chinese and Western Medicine (Henan Province), Zhengzhou, China; 3 Henan Province Hospital of Traditional Chinese Medicine (TCM), Zhengzhou, China; 4 The Affiliated Cancer Hospital of Zhengzhou University and Henan Cancer Hospital, Zhengzhou, China; 5 Centre for Evidence-Based Chinese Medicine, Beijing University of Chinese Medicine, Beijing, China

**Keywords:** population screening, esophageal cancer (EC), nomogram, questionnaire-based screening, risk stratification

## Abstract

**Background:**

Early detection of esophageal cancer (EC) is an effective strategy for reducing mortality. This study aims to construct a predictive nomogram based on a questionnaire survey to assess the risk of EC in the Chinese population and achieve risk stratification.

**Methods:**

To ensure and verify the performance of the nomogram, we have established partnerships with 45 research institutions in 10 provinces in China. Our study comprised a total of 4016 participants (3423 participants were considered for the development and internal validation of the nomogram, while 593 participants were considered for the independent external validation). Data were collected via standardized questionnaires, and relevant predictors were screened using a least absolute shrinkage and selection operator (LASSO) and multivariate logistic regression analysis. Finally, 10 core predictive factors were selected based on clinical significance and predictive performance to construct the predictive nomogram. The performance of the nomogram was evaluated using the area under the receiver operating characteristic curve (AUC) and calibration curves, while its clinical benefit was assessed using decision curve analysis (DCA) and clinical impact curves (CIC).

**Results:**

In the training cohort, using LASSO regression technology, multivariate logistic regression analysis, and consideration of the clinical significance of variables, 10 predictive factors were ultimately selected to construct a nomogram, including nutritional status, preference for hot food, rate of eating, fruit intake, preference for pickled food, preference for hard food, sex, vegetable intake, age, and cooking oil intake. The nomogram performed well in both the internal validation (corrected AUC = 0.806) and external validation datasets (corrected AUC = 0.843). The calibration curve showed that the nomogram was in good agreement with the observed outcomes. The results of DCA and CIC show that our nomogram demonstrated favorable clinical consistency.

**Conclusion:**

This study developed a nomogram, which has been proven to be a convenient, economical, and accurate tool for effectively predicting the risk of developing EC in individual Chinese populations, enabling risk stratification, and serving as a pre-screening tool prior to endoscopic screening for EC.

## 1 Introduction

China is the country with the highest burden of esophageal cancer (EC) globally. According to the 2022 Global Cancer Statistics, China leads the world in both new EC cases and EC-related deaths (1, 2). The high incidence and mortality rates of EC pose a significant challenge to China’s healthcare system (3). Screening high-risk populations of EC, detecting EC and precancerous lesions at an early stage, and intervening proactively play a crucial role in reducing EC mortality rates (4, 5). Currently, upper gastrointestinal endoscopy is the gold standard for screening and diagnosing EC (6). However, due to China’s large population and limited medical resources, it is not yet feasible to screen the entire population for EC through upper gastrointestinal endoscopy. Therefore, it is necessary to develop pre-screening tools to proactively identify high-risk groups for EC prior to endoscopy.

In China, a series of national-level cancer screening and early diagnosis and treatment programs have been implemented for many years. The prevention and control of EC have achieved initial success, and relevant data have provided conclusive evidence that upper gastrointestinal endoscopic screening can effectively reduce the risk of EC mortality (7). However, due to China’s large population, there is a relative shortage of endoscopic diagnostic and therapeutic resources (including equipment and specialized physicians), and the population has low compliance with invasive endoscopic examinations. As a result, the current screening system is still unable to meet the public health needs for early EC screening (8). It is worth noting that some studies have shown that a “one-step” endoscopic screening strategy without preliminary screening measures has no significant effect on reducing the incidence and mortality of EC (9, 10). Therefore, developing non-invasive risk stratification methods prior to endoscopic examinations is necessary and will help improve the efficiency and effectiveness of EC endoscopic screening. In China, multiple predictive nomograms have been developed to estimate the risk of EC, with AUC values ranging from 0.681 to 0.843 (11–15). However, most of these nomograms were developed based on the population characteristics of a single high-risk EC region, and only one studied underwent external validation (15). A 10-year EC absolute risk prediction nomogram developed in a study may be particularly useful for populations in areas with low EC risk (16). These factors may limit the nomograms’ generalizability and practicality. Constructing predictive nomograms by broadly incorporating population characteristics from different EC risk regions through a multi-center survey approach and conducting rigorous external validation is a feasible path to enhance the performance and generalizability of nomograms, which will further improve the effectiveness of early EC screening. Therefore, there is an urgent need to construct a widely applicable, convenient, economical, and accurate EC risk prediction model that can serve as a pre-screening tool for identifying high-risk individuals for EC in China.

To ensure the validity and reliability of the predictive nomogram, the research team conducted a pioneering multicenter case-control study. The study collaborated with 45 research institutions across 10 provinces in China to develop a predictive nomogram using cohorts of EC and non-EC participants from different regions, and evaluated the performance of nomogram through internal and external validation. By leveraging the power of diagnostic modeling, this nomogram will provide an effective, personalized method for screening EC for the general population in China, particularly in economically underdeveloped regions or primary healthcare facilities lacking advanced diagnostic equipment, thereby further improving the early diagnosis rate of EC.

## 2 Materials and methods

### 2.1 Ethics approval

This study was approved by the Ethics Committee of Henan Provincial Hospital of Traditional Chinese Medicine (Approval Number: Hospital Ethics Review No. (1337-01)) and conducted in accordance with the ethical standards outlined in the Declaration of Helsinki. All participants in this study provided written informed consent.

### 2.2 Study population

This study included 4,018 participants (including 2,027 EC participants and 1,991 non-EC participants) who visited 45 research institutions in 10 provinces across the country between June 2020 and March 2021. Currently, multiple predictive models have been developed for EC screening, demonstrating promising efficacy in both high- and low-risk regions. However, a specialized, robustly optimized predictive tool remains lacking for the intermediate-risk EC scenario—a setting frequently overlooked in practical screening efforts yet holding significant public health implications. Therefore, based on the characteristics of existing data, this study selected 3,425 participants (including 1,732 EC patients and 1,693 non-EC patients) from nine provinces in China (excluding Gansu Province) covering high-, medium-, and low-risk regions for EC. This cohort was used for nomogram development and internal validation. Concurrently, 593 participants (including 295 EC patients and 298 non-EC patients) from Gansu Province (a medium-risk region for EC) were designated as an independent external validation cohort to comprehensively assess the nomogram’s generalization capability and clinical applicability in real-world medium-risk settings (17). Since EC patients in China are predominantly esophageal squamous cell carcinoma (ESCC) patients, accounting for more than 90% of cases (18), we only included ESCC patients in this study. All participants had received definitive pathological diagnoses and were further divided into two groups: EC group and non-EC control group.

### 2.3 Inclusion and exclusion criteria

Inclusion criteria for EC group participants: 1) Meet the pathological diagnostic criteria for ESCC; 2) Participants provide informed consent for the study and sign the informed consent form.

Inclusion criteria for non-EC control group participants: 1) Reside in the same area as EC group participants (area scope: administrative village) or are non-EC family members of EC group participants; 2) Participants provide informed consent for the study and sign the informed consent form.

Exclusion criteria for both EC and non-EC groups: 1) History of mental disorders; 2) Patients with liver, kidney, heart, or hematopoietic system diseases; 3) Participants who were unable to provide complete information or complete the questionnaire during the survey; 4) Patients with malignant tumors in other parts of the body (excluding primary EC that has metastasized to other sites).

### 2.4 Data collection

This study utilized a survey questionnaire developed by the research team. The questionnaire content was developed by multiple clinical experts specializing in oncology, who referenced the 2020 Chinese Society of Clinical Oncology (CSCO) “Guidelines for the Diagnosis and Treatment of Esophageal Cancer” (19) and related literature, and combined clinical practice and survey priorities to create the questionnaire independently (20–27). Through the steps of drafting the initial questionnaire, conducting a pilot survey, holding multiple expert discussions, and revising the questionnaire repeatedly, the final version of the questionnaire was developed, which is comprehensive in content, well-structured, and highly practical. All variables were based on the questionnaire items and met any of the following criteria: 1) baseline demographic information; 2) biological plausibility for predicting EC risk (28); 3) reported in published studies on EC risk factors (29–32). The final variables included: 1) baseline demographic information: sex, age, nation, marital status (MS), living environment (LE), geographical characteristic (GC), educational attainment (EA) and careers); 2) Past medical history: health status (HS), history of esophageal disease (HOED), history of gastritis (HOG), history of gastric and duodenal ulcers (HGDU), helicobacter pylori infection (HPI), human papillomavirus (hpv) infection (HPV), history of oral disease (HOOD), history of hypertension (HOH) and history of diabetes (HOD); 3) Personal and family history: family history of esophageal cancer (FHOEC), years of smoking (YOS), years of drinking (YOD) and history of pesticide exposure (HOPE); 4) Lifestyle: labor intensity (LI), nutritional status (NS), oral cleanliness (OC) and type of drinking water (TODW); 5) Dietary habits: salt intake (SI), preference for hot food (PFHF), preference for hard food (PFHFS), preference for rough food (PFRF), preference for pickled food (PFPF), preference for smoked and grilled food (PFSG), ingestion of moldy food (IOMF), preference for soybean product (PFSP), rate of eating (ROE), eat and drink unreasonably (EADU), vegetable intake (VI), fruit intake (FI), tea intake (TI), garlic intake (GI), chili intake (CI), cooking oil intake (COI) and meat intake (MI). Specific information on all variables is shown in **Table 1**. All investigators had relevant medical backgrounds and had undergone pre-survey training and rigorous screening. During the survey interviews, the criteria for judging the items in the questionnaire were strictly consistent, and the external validation dataset was independently collected.

**Table 1:** Characteristics of the participants who enrolled in our study.

Variables^*^	Total participants (n = 4016)	Training cohort (n = 3423)			External validation cohort (n = 593)			*P*-value
		EC participants (n = 1730)	Non-EC participants (n = 1693)	*P*-value^*^	EC participants (n = 295)	Non-EC participants (n = 298)	*P*-value^#^	0.755
Sex, n(%)				<0.001			<0.001	0.570
Female	1371 (34.14%)	433 (25.03%)	729 (43.06%)		42 (14.24%)	167 (56.04%)		
Male	2645 (65.86%)	1297 (74.97%)	964 (56.94%)		253 (85.76%)	131 (43.96%)		
Age, years, M ± SD	64.17 ± 10.57	65.75 ± 9.14	62.94 ± 12.16	<0.001	63.92 ± 8.63	62.20 ± 8.94	0.019	<0.001
HOED, n(%)				<0.001			0.902	0.001
No	3941 (98.13%)	1669 (96.47%)	1679 (99.17%)		295 (100%)	298 (100.00%)		
Yes	75 (1.87%)	61 (3.53%)	14 (0.83%)		0 (0%)	0 (0.00%)		
FHOEC, n(%)				0.007			0.902	<0.001
No	3799 (94.60%)	1601 (92.54%)	1605 (94.80%)		295 (100%)	298 (100.00%)		
Yes	217 (5.40%)	129 (7.46%)	88 (5.20%)		0 (0%)	0 (0.00%)		
YOS, years, M ± SD	13.96 ± 19.51	16.08 ± 20.11	9.74 ± 17.77	<0.001	25.93 ± 19.07	13.69 ± 19.07	<0.001	<0.001
YOD, years, M ± SD	10.65 ± 17.75	13.58 ± 18.97	6.86 ± 15.47	<0.001	17.99 ± 19.13	7.96 ± 15.73	<0.001	<0.001
NS, n(%)				<0.001			<0.001	<0.001
Poor	404 (10.06%)	343 (19.83%)	52 (3.07%)		8 (2.71%)	1 (0.34%)		
Fair	2264 (56.37%)	1151 (66.53%)	953 (56.29%)		109 (36.95%)	51 (17.11%)		
Good	1348 (33.57%)	236 (13.64%)	688 (40.64%)		178 (60.34%)	246 (82.55%)		
PFHF, n(%)				<0.001			<0.001	<0.001
No	2051 (51.07%)	754 (43.58%)	1203 (71.06%)		31 (10.51%)	63 (21.14%)		
Yes	1965 (48.93%)	976 (56.42%)	490 (28.94%)		264 (89.49%)	235 (78.86%)		
PFHFS, n(%)				<0.001			<0.001	<0.001
No	2558 (63.70%)	969 (56.01%)	1257 (74.25%)		94 (31.86%)	238 (79.87%)		
Yes	1458 (36.30%)	761 (43.99%)	436 (25.75%)		201 (68.14%)	60 (20.13%)		
PFRF, n(%)				0.359			0.902	<0.001
No	3757 (93.55%)	1592 (92.02%)	1572 (92.85%)		295 (100%)	298 (100.00%)		
Yes	259 (6.45%)	138 (7.98%)	121 (7.15%)		0 (0%)	0 (0.00%)		
PFPF, n(%)				<0.001			<0.001	0.021
None	488 (12.15%)	216 (12.49%)	223 (13.17%)		25 (8.47%)	24 (8.05%)		
Dislike	1220 (30.38%)	466 (26.94%)	544 (32.13%)		72 (24.41%)	138 (46.31%)		
Medium	1328 (33.07%)	452 (26.13%)	627 (37.03%)		142 (48.14%)	107 (35.91%)		
Like	980 (24.40%)	596 (34.45%)	299 (17.66%)		56 (18.98%)	29 (9.73%)		
PFSG, n(%)				<0.001			0.007	<0.001
No	3800 (94.62%)	1600 (92.49%)	1614 (95.33%)		288 (97.63%)	298 (100.00%)		
Yes	216 (5.38%)	130 (7.51%)	79 (4.67%)		7 (2.37%)	0 (0.00%)		
IOMF, n(%)				<0.001			0.030	<0.001
No	3784 (94.22%)	1565 (90.46%)	1631 (96.34%)		290 (98.31%)	298 (100.00%)		
Yes	232 (5.78%)	165 (9.54%)	62 (3.66%)		5 (1.69%)	0 (0.00%)		
PFSP, n(%)				0.391			0.013	0.054
Dislike	1159 (28.86%)	498 (28.79%)	510 (30.12%)		62 (21.02%)	89 (29.87%)		
Like	2857 (71.14%)	1232 (71.21%)	1183 (69.88%)		233 (78.98%)	209 (70.13%)		
ROE, n(%)				<0.001			<0.001	<0.001
Slow	727 (18.10%)	286 (16.53%)	339 (20.02%)		36 (12.20%)	66 (22.15%)		
Medium	1937 (48.23%)	691 (39.94%)	1011 (59.72%)		57 (19.32%)	178 (59.73%)		
Quick	1352 (33.67%)	753 (43.53%)	343 (20.26%)		202 (68.47%)	54 (18.12%)		
VI, n(%)				<0.001			<0.001	<0.001
None	19 (0.47%)	12 (0.69%)	5 (0.30%)		2 (0.68%)	0 (0.00%)		
Dislike	353 (8.79%)	189 (10.92%)	82 (4.84%)		69 (23.39%)	13 (4.36%)		
Medium	1552 (38.65%)	705 (40.75%)	592 (34.97%)		124 (42.03%)	131 (43.96%)		
Like	2092 (52.09%)	824 (47.63%)	1014 (59.89%)		100 (33.90%)	154 (51.68%)		
FI, n(%)				<0.001			<0.001	0.002
None	181 (4.51%)	134 (7.75%)	41 (2.42%)		6 (2.03%)	0 (0.00%)		
Dislike	1157 (28.81%)	602 (34.80%)	399 (23.57%)		119 (40.34%)	37 (12.42%)		
Medium	1558 (38.80%)	620 (35.84%)	684 (40.40%)		109 (36.95%)	145 (48.66%)		
Like	1120 (27.88%)	374 (21.62%)	569 (33.61%)		61 (20.68%)	116 (38.93%)		
COI, every day, n(%)				0.045			0.584	<0.001
Less than 25g	1048 (26.10%)	533 (30.81%)	468 (27.64%)		23 (7.80%)	24 (8.05%)		
25-50g	2333 (58.09%)	904 (52.25%)	915 (54.05%)		254 (86.10%)	260 (87.25%)		
More than 50g	635 (15.81%)	293 (16.94%)	340 (20.08%)		18 (6.10%)	14 (4.70%)		

*All variables were based on the questionnaire items and met any of the following criteria: 1) baseline demographic information; 2) biological plausibility for predicting EC risk; 3) reported in published studies on EC risk factors. Categorical variables are expressed as frequency and percentage (n, %), Continuous variables are expressed as mean ± standard deviation (M ± SD).

*The P-value refers to the comparison between EC participants and non-EC participants in the training cohort.

#The P-value refers to the comparison between EC participants and non-EC participants in the external validation cohort.

The P-value refers to the comparison between the training cohort and the external validation cohort.

### 2.5 Nomogram construction

To screen variables and minimize nomogram complexity, this study first employed LASSO regression analysis. Through 10-fold cross-validation and based on the minimum standard error criterion, the optimal penalty parameter λ (lambda) for the LASSO was determined in the training cohort. An increase in the penalty parameter causes the coefficients of weaker predictive factors to shrink to zero, thereby screening out the most predictive variables (33). This study selected the predictive covariates corresponding to the minimum λ value within one standard deviation (λ.1se) for subsequent analysis. The variables screened by LASSO regression were included in a multivariate logistic regression analysis (significance level set at p < 0.05) to identify independent predictors (34). Given the large number of independent predictive factors retained after the multivariate analysis, a backward variable selection method based on changes in the AUC value was further applied to refine the set, ultimately identifying 5 key predictive factors. Furthermore, 5 additional variables were included based on three criteria: significant clinical relevance, clinical accessibility, and ease of quantification (see **Supplementary File S3** for details). Together, these form the final set of 10 independent predictors. Based on this, a nomogram was constructed to predict the risk of EC occurrence. In the nomogram, each independent risk factor is assigned a corresponding score based on its weight. By summing the scores of all risk factors, their total score can be calculated to predict the probability of EC occurrence (35). LASSO regression analysis was implemented using the “glmnet” package in RStudio software, while the construction of the nomogram was completed using the “rms” package.

### 2.6 Statistical analysis

Continuous variables are expressed as mean ± standard deviation (M ± SD). Categorical variables are expressed as frequency and percentage (n, %), with multi-categorical variables further divided into unordered categorical variables (e.g., living environment, careers, etc.) and ordered categorical variables (e.g., educational attainment, labor intensity, etc.). Differences between two groups for continuous variables and ordered categorical variables were analyzed using the Mann-Whitney U test, while differences between groups for dichotomous variables and unordered categorical variables were analyzed using the Pearson chi-square (χ²) test. Multivariate logistic regression analysis was used to identify independent predictors of EC occurrence. Based on the results of the multivariate logistic regression analysis, a predictive receiver operating characteristic (ROC) curve was constructed using the “rms” package in RStudio software (version 4.4.1). The nomogram’s discriminatory ability was assessed by calculating the area under the curve (AUC) and its 95% confidence interval (95% CI). Calibration curves were used to assess the consistency between the nomogram-predicted probability of EC occurrence and the actual observed probability. DCA was used to assess the clinical net benefit of the nomogram at different risk thresholds. Additionally, CIC were plotted to visually demonstrate how the nomogram’s predicted risk stratification affects the number of patients classified as high-risk at different thresholds, as well as the corresponding true positive and false positive case counts, thereby quantifying the nomogram’s potential impact in clinical applications. All statistical analyses were performed using RStudio software (version 4.4.1). Hypothesis tests were conducted using two-sided tests, with statistical significance defined as p < 0.05.

The development, internal validation, external validation, and reporting of the nomogram strictly adhered to the guidelines of the Transparent Reporting of Individual Prognosis or Diagnosis Multivariate Prediction Models (TRIPOD) initiative (36).

## 3 Results

### 3.1 Participant’s characteristics

**Figure 1:**
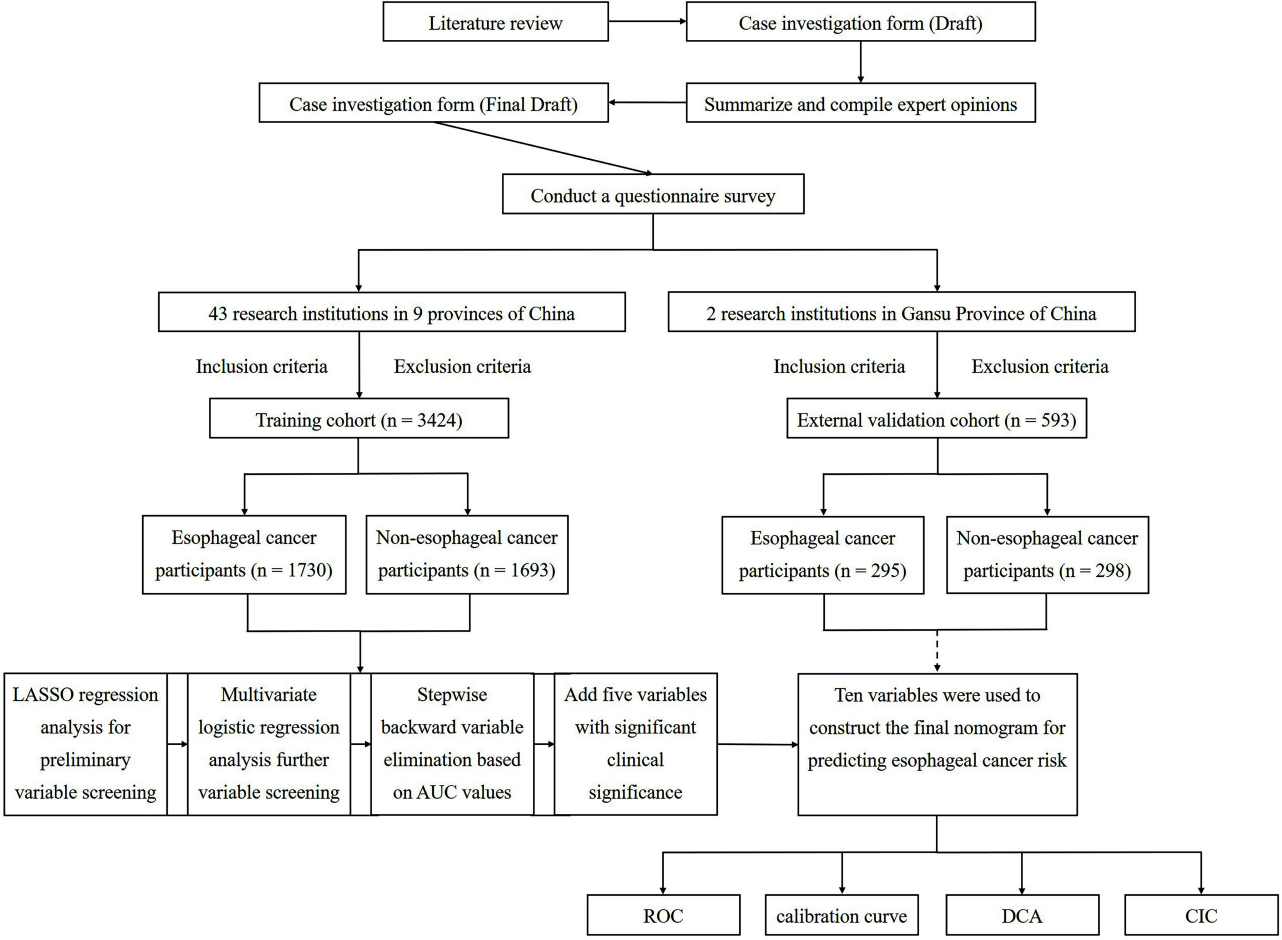
Research flowchart.

We first conducted a comprehensive screening for missing values across all included variables. Following this screening, only 2 patients were found to have missing information. We excluded these two participants with incomplete data. Thus, this study utilized 3,423 participants from the other nine provinces (including 1,730 EC participants and 1,693 non-EC participants) as the training cohort for the nomogram, and 593 participants from Gansu Province (including 295 EC participants and 298 non-EC participants) as an independent external validation cohort. **Figure 1** illustrates the flowchart of participant inclusion in the study. In the training cohort, there were 2,261 males (66.1%) and 1,162 females (33.9%), 1,413 participants living in non-rural areas (41.3%) and 2,010 participants living in rural areas (58.7%), with an average age of 64.36 ± 10.83 years (range 16–95 years). In the external validation cohort, there were 384 males (64.8%) and 209 females (35.2%), with 130 participants (21.9%) living in non-rural areas and 463 participants (78.1%) living in rural areas. The average age was 63.05 ± 8.84 years (range 41–86 years). All data for these two groups of participants, including baseline information, past medical history, personal and family history, lifestyle and dietary habits, are shown in **Table 1** (The full version is included in the **Supplementary Material S2**).

### 3.2 Prediction factor identification

**Figure 2:**
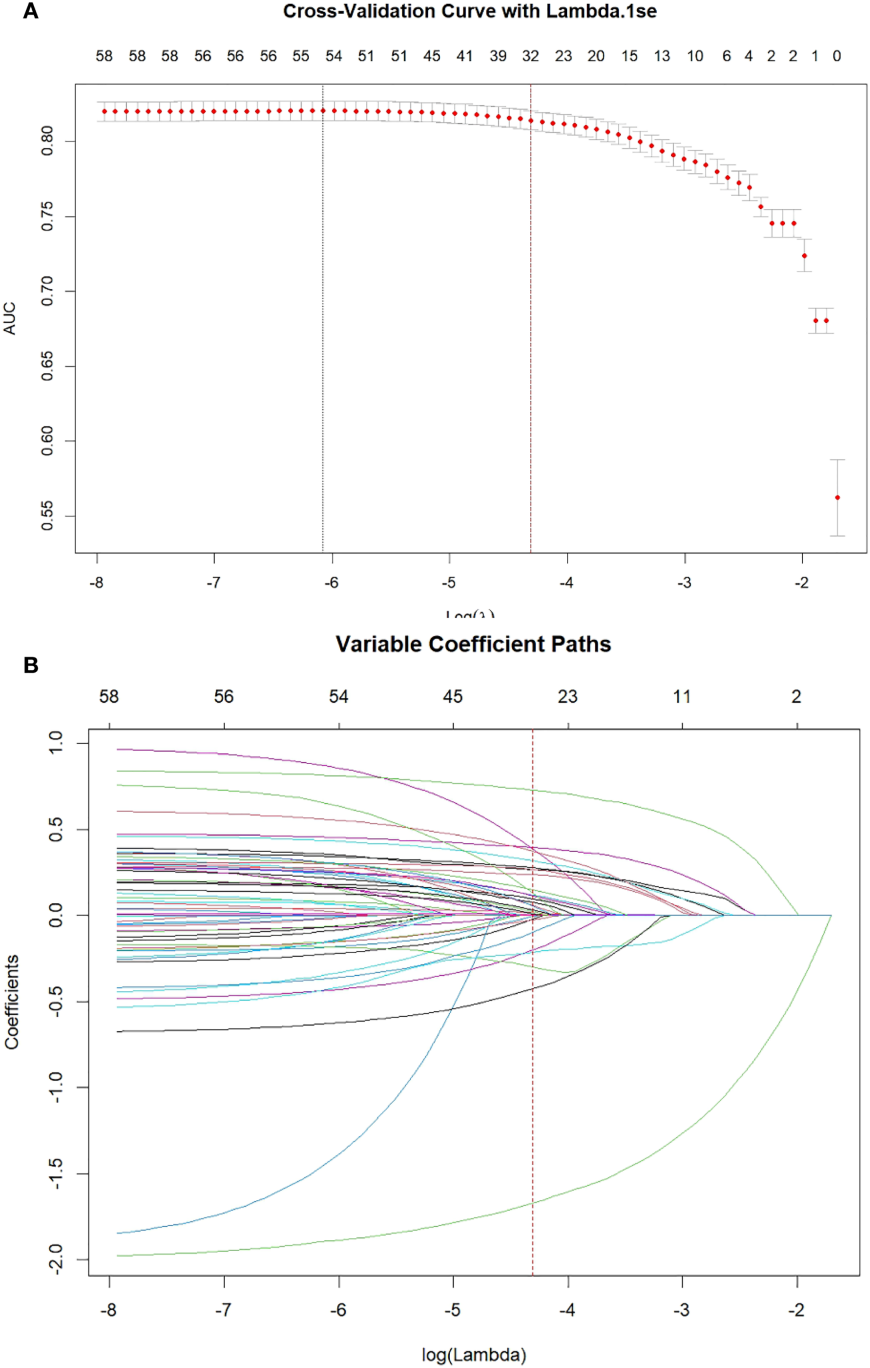
**(A)** Cross-Validation Plot. Identification of the optimal penalization coefficient lambda (λ) in the LASSO model with 10-fold cross-validation in training cohort. 25 variables had nonzero coefficients in the LASSO regression model based on the analysis of the training cohort. **(B)** Coefficient Path Plot. LASSO coefficient profiles of 42 features in training cohort. The trajectory of each EC-related features’ coefficient was observed in the LASSO coefficient profiles with the changing of the lambda in LASSO algorithm.

To identify potential important predictive indicators associated with the development of EC, this study applied LASSO regression analysis to the training cohort. The trends in the coefficients of each variable as a function of the regularization parameter λ are shown in **Figure 2A**. To select variables while ensuring nomogram predictive performance, the vertical axis of the cross-validation plot uses the AUC value, and the λ value corresponding to the minimum value of the biased likelihood binomial deviation plus one standard deviation (λ = 0.0134) is taken as the optimal solution. After LASSO regression screening, 25 variables were ultimately retained as important predictive factors for EC lesions, including sex, labor intensity, health status, age, history of esophageal disease, history of gastritis, nutritional status, years of drinking, salt intake, preference for hot food, preference for hard food, preference for soybean product, preference for pickled food, rate of eating, ingestion of moldy food, eat and drink unreasonably, vegetable intake, fruit intake, tea intake, cooking oil intake, meat intake, history of pesticide exposure, living environment, marital status and careers.

**Figure 3:**
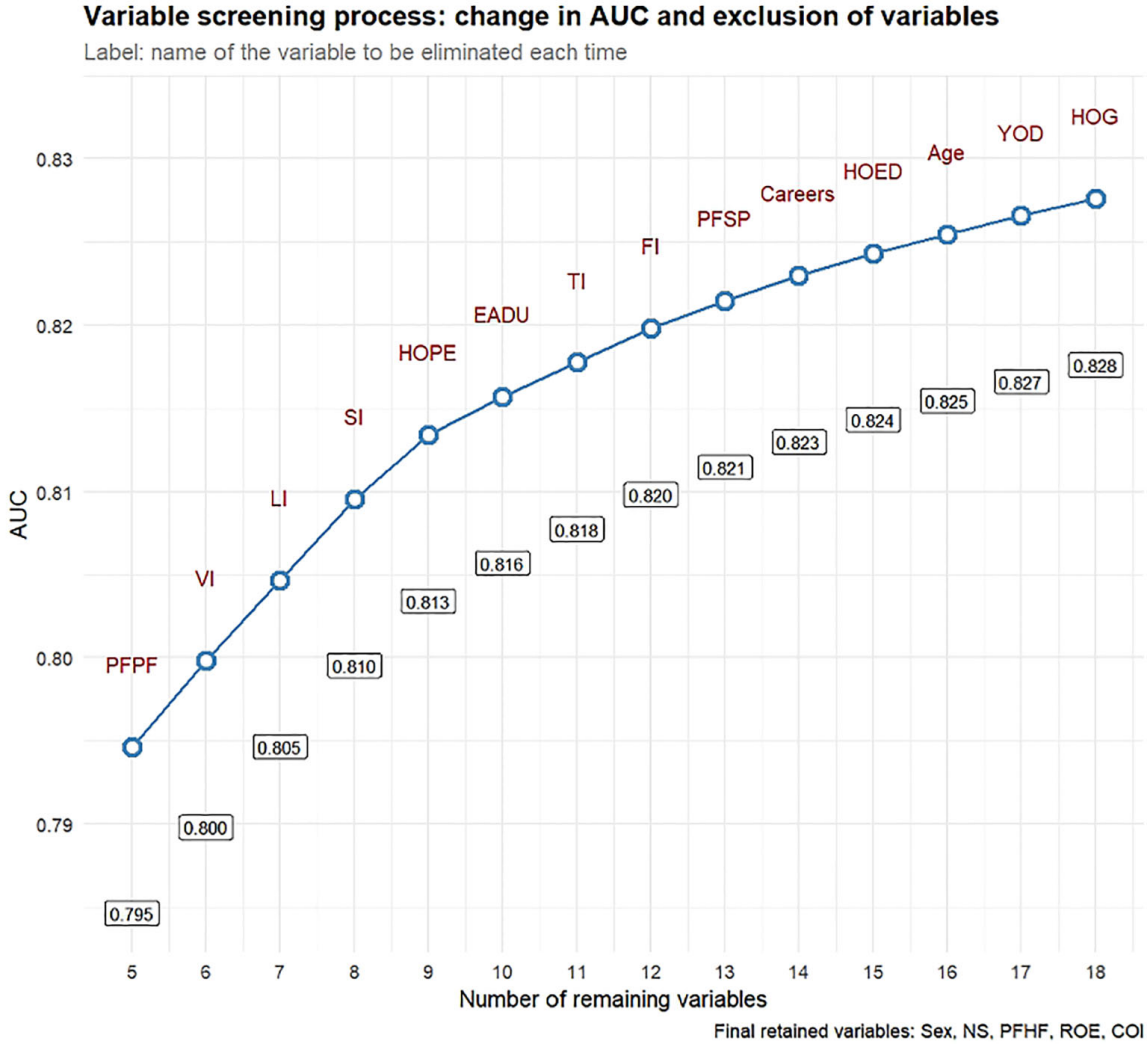
Detailed diagram of reverse stepwise elimination of variables. Based on changes in the AUC value, the nomogram model was repeatedly fitted and the variables with the smallest contribution to the AUC value were successively eliminated, ultimately yielding the final 5 important predictors: nutritional status, preference for hot food, rate of eating, sex, and cooking oil intake.

The above 25 variables were selected as candidate variables for multivariate logistic regression analysis. After stepwise regression analysis and likelihood ratio testing, six variables, including health status, meat intake, preference for hard food, ingestion of moldy food, living environment, and marital status, were excluded (*p* ≥ 0.05). The remaining 19 variables were all statistically significant (*p* < 0.05) (**Table 2**). To further screen variables and improve nomogram simplicity, we adopted a backward stepwise elimination strategy based on changes in the AUC value: repeatedly fitting the nomogram and sequentially eliminating variables with the smallest contribution to the AUC value until the nomogram retained the final 5 predictors: nutritional status, preference for hot food, eating rate, sex, and cooking oil intake (**Figure 3**). Subsequently, considering clinical practical significance, the 5 variables fruit intake, preference for pickled food, preference for hard food, vegetable intake, and age were included in the nomogram. Ultimately, the 10 variables comprising the final 5 retained variables and the 5 clinically significant variables were identified as core predictive factors for EC lesion occurrence and will be used for the creation of the nomogram.

**Table 2:** Multivariable analysis of risk factors for developing esophageal cancer.

Variables	Degree of freedom (Df)	Deviance	*P*-value
Sex	1	125.06	0.0000
LI	3	71.09	0.0000
Age	1	38.77	0.0000
HOED	1	28.34	0.0000
HOG	1	35.09	0.0000
NS	2	361.72	0.0000
YOD	1	49.88	0.0000
SI	2	106.67	0.0000
PFHF	1	141.01	0.0000
PFSP	1	37.19	0.0000
PFPF	3	34.68	0.0000
ROE	2	90.23	0.0000
EADU	1	12.81	0.0000
VI	3	31.38	0.0000
FI	3	17	0.0000
TI	2	9.56	0.0100
COI	2	55.44	0.0000
HOPE	1	9.59	0.0000
Careers	1	10.3	0.0000

### 3.3 Nomogram construction

**Figure 4:**
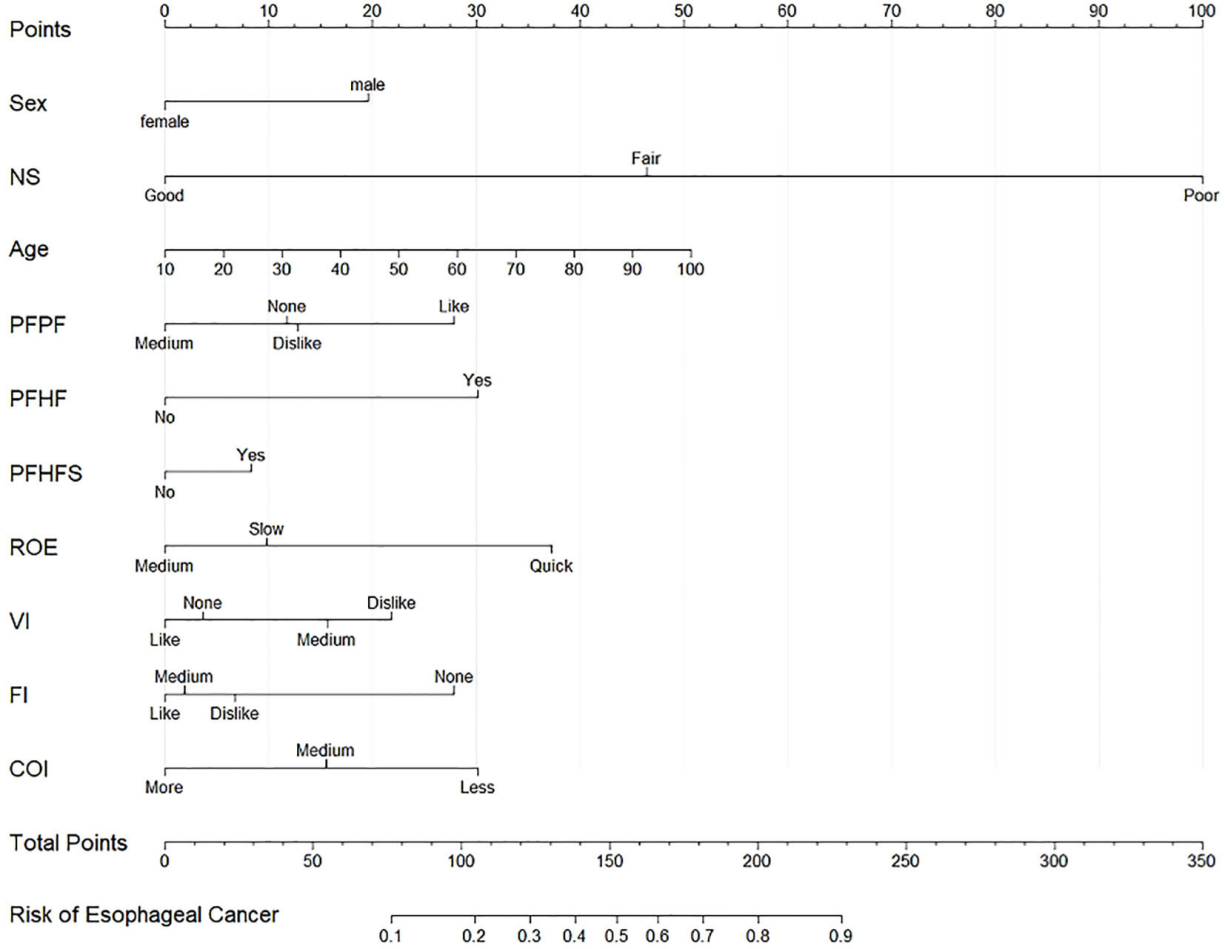
A nomogram for predicting the risk of esophageal cancer in individual participants. Add the scores for each feature to obtain the total score, and draw a vertical line on the total score to obtain the corresponding “esophageal cancer risk.” NS, nutritional status; PFPF, preference for pickled food; PFHF, preference for hot food; PFHFS, preference for hard food; ROE, rate of eating; VI, vegetable intake; FI, fruit intake; COI, cooking oil intake.

Subsequently, based on the 10 independent predictive factors identified above, a predictive nomogram was constructed to predict the probability of an individual developing EC (**Figure 4**). In this nomogram (**Figure 4**), the calculation process for individual risk scores is as follows: 1) Variable scoring: Locate the specific value of the subject on the corresponding variable axis (e.g., age 50, male, etc.), and read the corresponding score for that variable on the “Points” axis (range: 0–100 points). 2) Total Score Calculation: Add all individual variable scores to obtain the total score (Total Points). 3) Risk Probability Prediction: Locate the total score on the “Risk of Tumor Lesion” axis at the bottom to read the predicted probability of EC lesions for the individual (The complete formula is provided in **Supplementary File S1**).

### 3.4 Assessment of predictive accuracy of the nomogram

**Figure 5:**
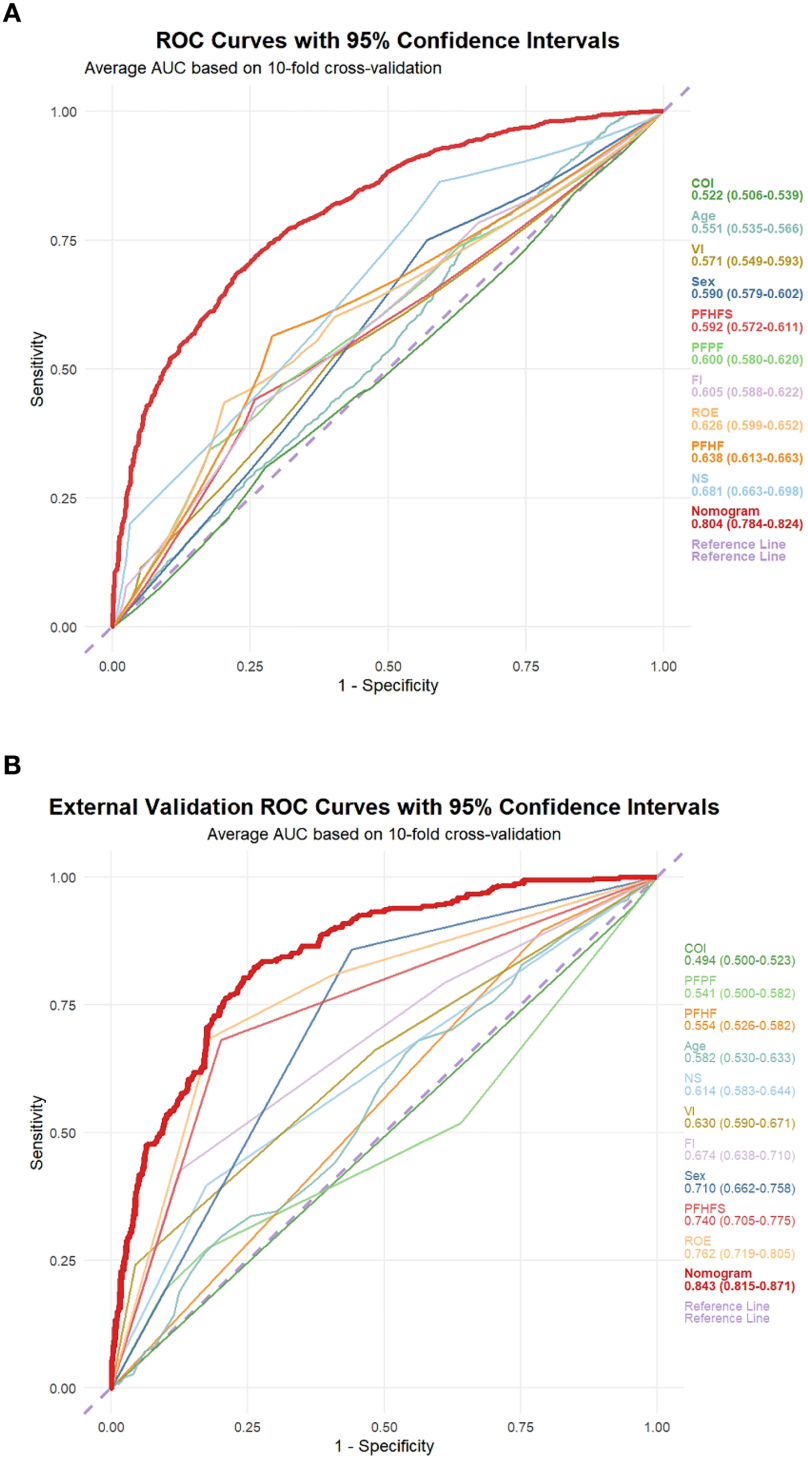
ROC curves for predicting risk of developing esophageal cancer. The receiver operating characteristics (ROC) curves of the nomogram model and single independent predictor in the training cohort **(A)** and external validation cohort **(B)**. The nomogram model displayed reliable diagnostic performance for prediction of developing esophageal cancer in both the training cohort (AUC, 0.804) and external validation cohort (AUC, 0.843).

**Figure 6:**
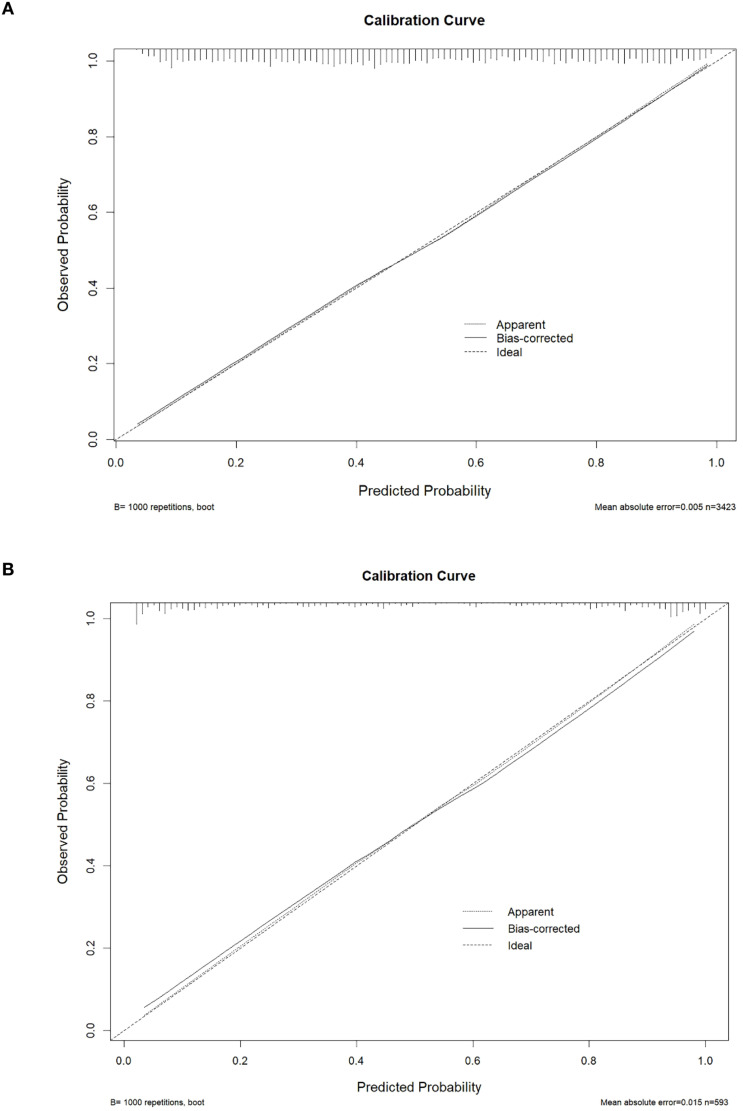
The calibration curve of the nomogram in the training and external validation cohort. The calibration plot was applied to compare the agreement between actual and predicted probability of developing esophageal cancer in the training cohort **(A)** and external validation cohort **(B)**.

**Figure 7:**
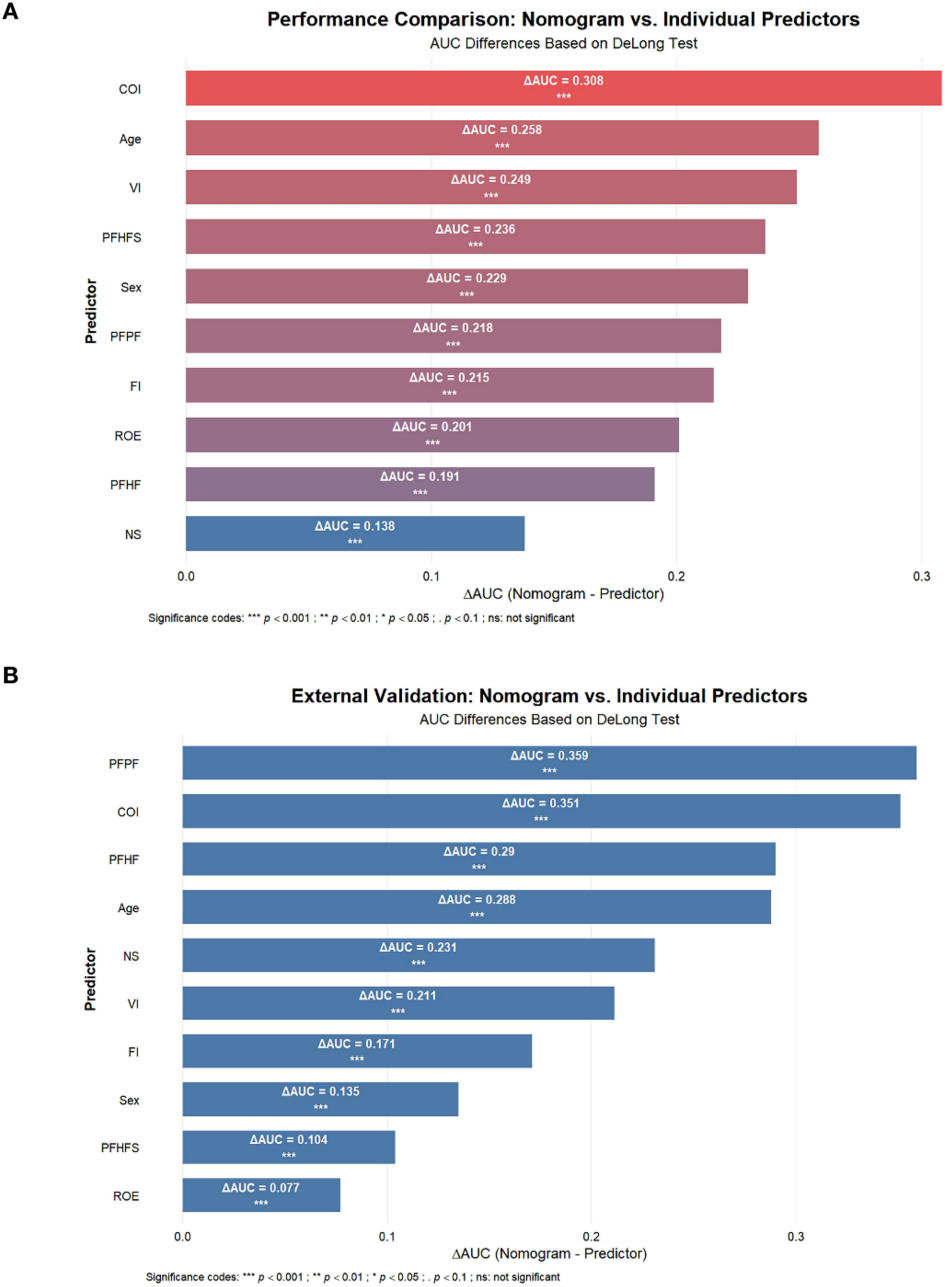
The result of Delong test. Delong test between nomogram model and single independent predictor in the training **(A)** and external validation cohort **(B)**.

To assess the discriminatory ability of the nomogram, this study calculated the area under the receiver operating characteristic (ROC) curve (AUC) value. Through 10-fold cross-validation, the nomogram achieved an AUC of 0.804 (95% CI: 0.784–0.824) in the training cohort (**Figure 5A**). Internal validation using the Bootstrap method revealed an original AUC of 0.811, with a corrected AUC of 0.806 and an optimism bias of 0.005 (**Table 3**). In the external validation cohort, the nomogram’s AUC was 0.844 (95% CI: 0.795–0.894) (**Figure 5B**), with a corrected AUC of 0.843 calculated using the Bootstrap method and an optimism bias of 0 (**Table 3**). These results indicate that the nomogram has good predictive capability. Additionally, the calibration curves were plotted to assess the predictive calibration of the nomogram. The calibration curves for both the training cohort (**Figure 6A**) and the external validation cohort (**Figure 6B**) showed that the predicted probabilities were highly consistent with the actual observed probabilities. The results of the DeLong test indicated that the predictive performance of the nomogram model was significantly superior to any single independent predictor in both the training cohort (**Figure 7A**) and the external validation cohort (**Figure 7A**).

**Table 3:** Internal and external validation of nomogram model by the bootstrap method.

Measure	Internal validation: nomogram performance			External validation: nomogram performance		
	Original value	Optimism	Corrected	Original value	Optimism	Corrected
AUC	0.811	0.005	0.806	0.844	0.000	0.843
*R* ^2^	0.372	0.011	0.361	0.414	-0.002	0.415
Brier score	0.177	-0.002	0.180	0.168	0.000	0.168
Slope	1	0.025	0.975	1.345	0.015	1.331

### 3.5 Clinical application value of nomogram

**Figure 8:**
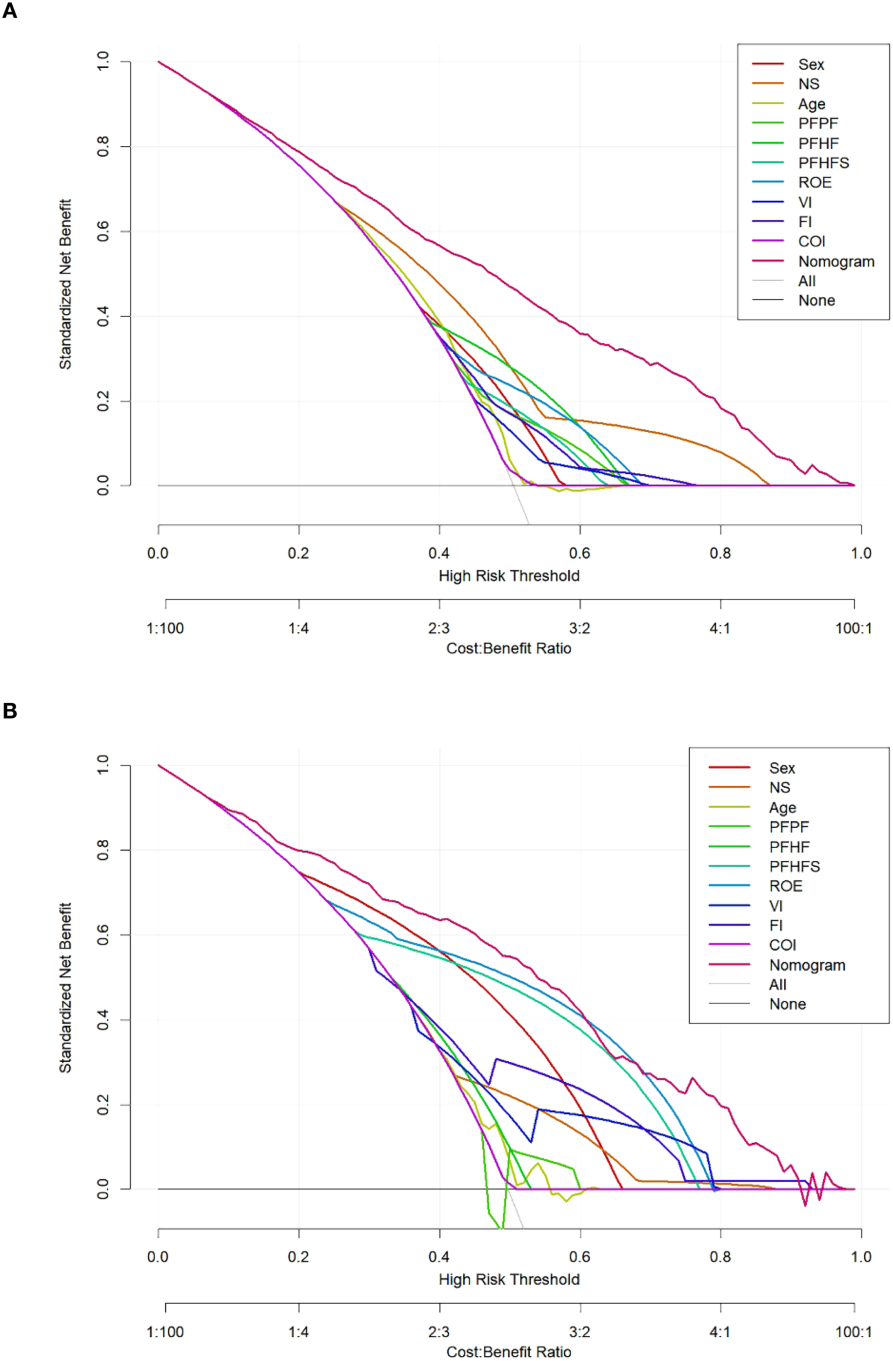
The decision curve analysis (DCA) of the nomogram prediction in the training and external validation cohort. DCA for the prediction nomogram model and single independent predictor in the training cohort **(A)** and external validation cohort **(B)**.

**Figure 9:**
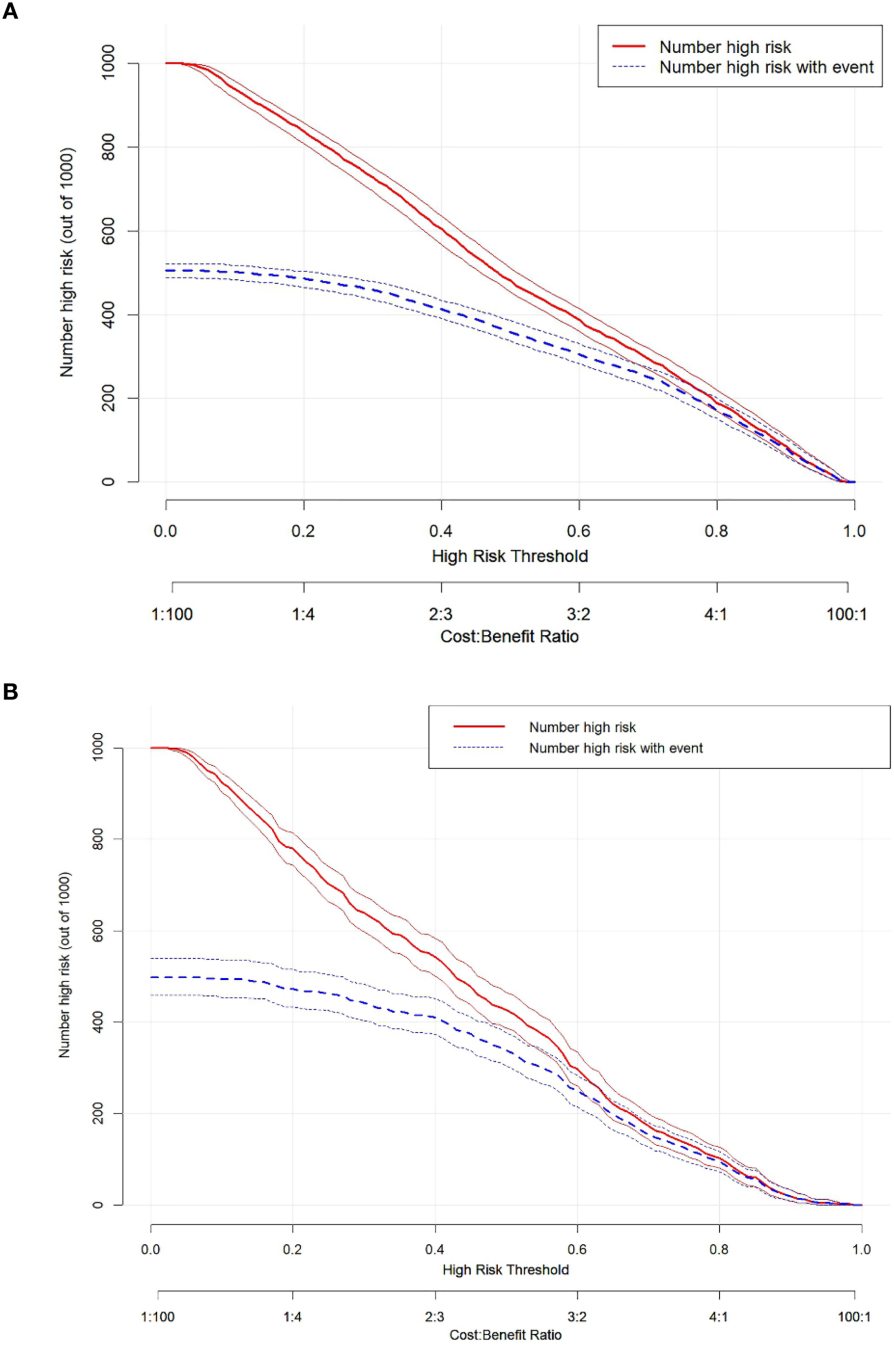
The clinical impact curve (CIC) of the nomogram prediction in the training and external validation cohort. CIC for the prediction nomogram model and single independent predictor in the training cohort **(A)** and external validation cohort **(B)**.

To assess the clinical utility of the nomogram, this study conducted DCA and plotted a CIC. As shown in **Figure 8A**, the horizontal axis of the DCA plot represents the threshold probability, and the vertical axis represents the net benefit. The analysis results showed that, in the training cohort, the net benefit of predicting the risk of EC using the nomogram was higher than that of any single predictor; a similar trend was observed in the external validation cohort (**Figure 8B**). The clinical impact curve further demonstrated that risk stratification based on the nomogram exhibited good clinical applicability in both the training cohort (**Figure 9A**) and the external validation cohort (**Figure 9B**), indicating that the nomogram can effectively identify individuals with different risk levels.

### 3.6 The nomogram constructed from 5 core predictors

We constructed a simplified version of nomogram 2 based on 5 core predictors and plotted corresponding ROC curves in the training cohort (AUC = 0.792, 95% CI: 0.778–0.806) and external validation cohort (AUC = 0.810, 95% CI: 0.768–0.852). Internal validation via Bootstrap sampling (original AUC = 0.795, corrected AUC = 0.790, optimism bias = 0.005) and external validation (original AUC = 0.807, corrected AUC = 0.806, optimism bias = 0.001) confirmed demonstrated that the simplified nomogram 2 possesses robust EC risk prediction capability. Although the full nomogram exhibits superior risk prediction performance, the simplified model still performs admirably. Furthermore, calibration curves plotted in both the training and external validation cohorts indicate high consistency between predicted and observed probabilities. DeLong tests further confirmed that the simplified nomogram 2 significantly outperformed any single predictor in both cohorts. DCA and CIC results demonstrated that this simplified nomogram 2 holds significant clinical application in both the training and external validation cohorts. Detailed results and analyses are presented in **Supplementary Material S1**.

## 4 Discussion

This study developed a nomogram model for predicting the risk of EC based on a training cohort. The nomogram demonstrated good predictive performance in the training cohort, internal validation using the Bootstrap method, and external validation. Our results indicate that nutritional status, preference for hot food, rate of eating, sex and cooking oil intake are important risk factors for EC. Calibration curve and ROC curve evaluation results confirm that the nomogram has high predictive accuracy and good discriminatory ability. Furthermore, DCA and CIC further demonstrate its good clinical application value. In summary, the nomogram can serve as a simple, accurate, and practical individualized risk assessment tool for preliminary screening of EC risk in the Chinese population.

A comprehensive understanding of the risk factors for EC is crucial for its prevention and for identifying high-risk populations requiring further endoscopic screening. The multivariate analysis in this study revealed that a total of 19 predictive factors (e.g., sex, labor intensity, health status, age, history of esophageal disease) were statistically significant (P < 0.05), consistent with previous studies (29, 30, 37–41). To identify core predictive factors closely associated with EC incidence and optimize the predictive nomogram model, this study employed a reverse stepwise elimination strategy based on changes in AUC contribution values, ultimately identifying 5 important predictive factors. Based on the predictive performance (AUC values) of individual variables on the ROC curve, the factors were ranked from highest to lowest as follows: nutritional status, preference for hot food, rate of eating, sex, cooking oil intake. Furthermore, considering clinical practicality, we included 5 additional predictive factors: fruit intake, preference for pickled food, preference for hard food, vegetable intake, age (42–44).

Our research findings and decisions are supported by previous studies. Numerous studies have shown that nutritional status is significantly associated with the occurrence and development of EC. Both deficiencies and excesses of specific nutrients can significantly influence the risk of EC onset; simultaneously, EC patients often have concomitant malnutrition, creating a bidirectional influence (37, 45–47). Currently, with the widespread knowledge of carcinogens and improved living standards, the influence of previously important EC risk factors (such as consuming moldy food, drinking untreated raw water, and excessive intake of pesticide residues) is gradually diminishing (48, 49). In contrast, poor dietary habits have emerged as the primary risk factor for EC in China (50, 51). Therefore, this study incorporated dietary habits-related predictive factors such as preference for hot food, preference for hard food, preference for pickled food and rate of eating into the predictive nomogram. Poor dietary habits can significantly increase susceptibility to esophageal mucosal damage. For example, long-term consumption of rough, hard foods, a preference for hot foods, and a high-salt diet can all damage the esophageal mucosa (52). It is worth noting that this is also an important reason why squamous cell carcinoma accounts for 90% of esophageal cancer cases in Chinese people (37). Meta-analysis and case-control study data indicate that high-temperature dietary behavior (>65 °C) (including eating, drinking water, drinking tea, etc.) significantly increases the risk of esophageal squamous cell carcinoma (OR = 2.39, 95% CI: 1.71 - 3.33) (OR = 1.67, 95% CI: 1.25 - 2.24), while no significant association was observed for esophageal adenocarcinoma (OR = 0.78, 95% CI: 0.45 - 1.35) (31, 53). This difference may stem from the fundamental differences in the pathogenesis of the two histological subtypes. The esophagus is composed of the mucosal layer, submucosal layer, and muscular layer. Long-term consumption of excessively hot or coarse-textured foods, as well as abnormal eating behaviors (including eating too quickly or too slowly), can lead to repeated mechanical and thermal damage to the esophageal mucosa. This chronic stimulation can induce persistent inflammatory responses in the mucosal epithelium, leading to superficial ulcers. In a vicious cycle of “damage-repair,” genomic instability in epithelial cells increases, ultimately resulting in gene mutations that trigger carcinogenesis. The increased risk of EC associated with pickled foods (OR = 2.10, 95% CI: 1.64 - 2.69) and the underlying mechanisms have been reported in multiple studies (54). First, the high levels of nitrates and nitrites in pickled foods can be converted into N-nitroso compounds—a known carcinogen (IARC Group 1 carcinogen) (55); Second, the production process of pickled foods generates heterocyclic amines (HCAs) and polycyclic aromatic hydrocarbons (PAHs), which are carcinogens (56); Third, high concentrations of salt can directly damage the esophageal mucosa, leading to chronic esophagitis and increasing the risk of EC (57).

In contrast to the aforementioned risk factors, soluble dietary fiber, various vitamins, and minerals such as iron and magnesium have been shown to have a protective effect against EC (38, 58–61). Therefore, moderate intake of fruits and vegetables has been proven to be beneficial, as reported in previous studies (62, 63). Meta-analysis clearly shows that high intake of fresh vegetables and fresh fruits significantly reduces the risk of esophageal squamous cell carcinoma in the population (RR = 0.56, 95% CI: 0.45 - 0.69), (RR = 0.53, 95% CI: 0.44 - 0.64) (64). The findings of this study support the conclusions of previous research, namely that men have a significantly higher risk of developing EC than women. This difference may be attributed to the higher prevalence of unhealthy lifestyle habits (such as smoking, excessive alcohol consumption, and drinking hot tea) in the male population (65, 66). Additionally, the protective effect of estrogen in the pathogenesis of EC has been supported by biological evidence (67, 68). Given this, this study did not include smoking and alcohol consumption as predictive variables in the final nomogram, and more importantly, because the difficulty in categorizing the severity of these variables and the possibility that they may not be the primary factors contributing to EC in the Chinese population (37). Furthermore, as described in the literature, the risk of EC increases significantly with age, particularly after the age of 45 (69). Therefore, regular endoscopic screening is recommended for individuals aged 45–75 years (70).

It is worth noting that our research findings indicate that dietary oil intake is a protective factor against EC incidence. According to previous studies, plant oils rich in unsaturated fatty acids have a protective effect against EC incidence, while oils high in saturated fatty acids increase the risk of EC (32, 59, 70). However, cooking oils produce benzopyrene (IARC Group 1 carcinogen) and acrylamide (IARC Group 2A carcinogen) during high-temperature cooking processes such as frying and grilling. These compounds can damage esophageal cell DNA and induce mutations (71, 72). Therefore, selecting appropriate types of cooking oil (preferably plant-based oils) and reducing consumption of high-temperature cooked foods (such as fried and grilled foods) are important measures for effectively preventing the occurrence of EC.

This study has several advantages: first, the data sources are extensive and the model has undergone rigorous validation: Compared with most previous EC prediction models developed based on single-center, high-risk populations (11–15), this study leveraged a professionally designed questionnaire survey to integrate diverse population data from multiple provinces across China, covering high-, medium-, and low-risk regions for esophageal cancer. Furthermore, it incorporated case-control samples from multiple research centers. Consequently, the nomogram constructed in this study demonstrates significant advantages. Furthermore, the model underwent external validation in an independent medium-risk region sample, confirming its robust performance while enhancing its potential applicability and extrapolation capability across broader populations. Second, our nomogram is simple, easy to use, and highly adaptable: all predictive factors included in the final nomogram are easily obtainable, enabling the nomogram to be widely applied across various settings. Even in economically underdeveloped regions or primary healthcare facilities lacking advanced diagnostic equipment, physicians can conveniently use this nomogram for EC risk stratification. Third, the nomogram’s performance has been validated through multiple methods: through internal validation using the Bootstrap method (AUC = 0.806) and independent external validation on datasets with population heterogeneity (AUC = 0.843), the nomogram demonstrated robust discriminatory capability. Its performance metrics surpassed most EC prediction models reported in previous literature (AUC range: 0.681–0.880) (11–15), indicating strong potential for clinical application. Therefore, the nomogram demonstrates good generalization ability and applicability in the Chinese population.

This study has several limitations: First, while the case-control design employed in this study is efficient in exploratory disease risk modeling, it carries inherent methodological limitations. On one hand, EC patients may experience recall bias when recalling past lifestyle habits after diagnosis, as their disease state could influence their memory. On the other hand, both case and control recruitment relied on medical institutions, potentially failing to fully represent the general population and introducing selection bias. Although we endeavored to adjust for known confounders in our statistical analysis, these potential biases may still affect the precision of model estimates. Future prospective cohort studies are needed to further validate our model’s predictive efficacy. Secondly, external validation was conducted only on an independent sample from Gansu Province (a moderate-risk region for EC). While this preliminarily demonstrates the model’s generalizability in moderate-risk areas, its applicability in high-risk and low-risk regions remains unvalidated. Additionally, we observed a relatively uncommon phenomenon where the AUC value in external validation exceeded that in internal validation and the training cohort. This discrepancy may stem from the homogeneity of the external validation set. Therefore, while current results indicate the model’s strong generalizability, further external validation in both low- and high-risk EC regions is essential for a comprehensive assessment of its generalization capabilities. Third, model variable selection prioritized practicality: To enhance the acceptability and accessibility of this predictive tool in practice, this study prioritized easily obtainable medical history and lifestyle factors while excluding clinical laboratory indicators. However, certain known critical risk factors—such as detailed smoking and drinking classifications—were omitted due to challenges in precise quantification. While this approach enhances the tool’s practicality and simplicity, it may overlook other risk indicators highly correlated with EC incidence. Future research should consider incorporating a broader range of risk factors and integrating machine learning or deep learning algorithms to screen for variables with greater predictive value. This would enable the development of more specialized and precise EC risk prediction models tailored for healthcare professionals. Finally, sample size requires expansion: both the training cohort and external validation cohort need larger samples. Future optimization and validation using broader, more representative population data will enhance the model’s practical predictive performance.

## 5 Conclusions

In summary, we have developed a novel esophageal cancer (EC) risk predictive nomogram model. This nomogram is characterized by low cost, ease of use, and high predictive accuracy, making it an effective tool for EC risk stratification and initial screening prior to endoscopic screening. The nomogram demonstrated excellent discriminatory ability and good calibration in both internal and external validation, indicating its potential for application in EC early screening in a broader population. Further studies are needed to assess the actual efficacy of this nomogram in EC risk stratification in the real-world general population.

## Data Availability

The datasets used and/or analyzed during the current study are available from the corresponding author on reasonable request. Requests to access these datasets should be directed to zhengyl@hactcm.edu.cn.
