# Supplementary material for: Development and external validation of a nomogram for predicting the risk of developing esophageal cancer based on a questionnaire: a multicenter case-control study
Source: Front Oncol. 2025 Dec 4;15:1684561. doi: 10.3389/fonc.2025.1684561 (PMC12711463; doi:10.3389/fonc.2025.1684561)
Supplement: Supplementary file 1 [file Table1.docx]

Table1: Characteristics of the participants who enrolled in our study.

| Variables^*^ | Total participants  (n = 4016) | Training Cohort  (n = 3423) | | | External Validation Cohort  (n = 593) | | | *p*-value |
| --- | --- | --- | --- | --- | --- | --- | --- | --- |
|  |  | EC participants (n = 1730) | Non-EC participants (n = 1693) | *p*-value^*^ | EC participants (n = 295) | Non-EC participants (n = 298) | *p*-value^#^ | 0.755 |
| Sex, n(%) |  |  |  | <0.001 |  |  | <0.001 | 0.570 |
| Female | 1371 (34.14%) | 433 (25.03%) | 729 (43.06%) |  | 42 (14.24%) | 167 (56.04%) |  |  |
| Male | 2645 (65.86%) | 1297 (74.97%) | 964 (56.94%) |  | 253 (85.76%) | 131 (43.96%) |  |  |
| Age, years,  M ± SD | 64.17 ± 10.57 | 65.75 ± 9.14 | 62.94 ± 12.16 | <0.001 | 63.92 ± 8.63 | 62.20 ± 8.94 | 0.019 | <0.001 |
| Nation, n(%) |  |  |  | 0.544 |  |  | 0.196 | <0.001 |
| Other ethnic groups | 78 (1.94%) | 12 (0.69%) | 9 (0.53%) |  | 33 (11.19%) | 24 (8.05 %) |  |  |
| Han ethnic group | 3938 (98.06%) | 1728 (99.31%) | 1684 (99.47%) |  | 262 (88.81%) | 274 (91.95 %) |  |  |
| MS, n(%) |  |  |  | 0.001 |  |  | 1.000 | 0.119 |
| Married | 59 (1.47%) | 16 (0.92%) | 39 (2.30%) |  | 2 (0.68%) | 2 (0.67 %) |  |  |
| Unmarried | 3957 (98.53%) | 1714 (99.08%) | 1654 (97.70%) |  | 293 (99.32%) | 296 (99.33 %) |  |  |
| LE, n(%) |  |  |  | <0.001 |  |  | 0.146 | <0.001 |
| Cities | 1543 (38.42%) | 617 (35.66%) | 796 (47.02%) |  | 72 (24.41%) | 58 (19.46 %) |  |  |
| Rural | 2473 (61.58%) | 1113 (64.34%) | 897 (52.98%) |  | 223 (75.59%) | 240 (80.54 %) |  |  |
| GC, n(%) |  |  |  | 0.012 |  |  | 0.851 | <0.001 |
| More livable | 1902 (47.36%) | 709 (40.98%) | 623 (36.80%) |  | 284 (96.27%) | 286 (95.97 %) |  |  |
| Less livable | 2114 (52.46%) | 1021 (59.02%) | 1070 (63.20%) |  | 11 (3.73%) | 12 (4.03 %) |  |  |
| EA, n(%) |  |  |  | <0.001 |  |  | 0.294 | <0.001 |
| Other | 660 (16.43%) | 284 (16.42%) | 201 (11.87%) |  | 72 (24.41%) | 103 (34.56 %) |  |  |
| Elementary school education | 1727 (43.00%) | 762 (44.05%) | 642 (37.92%) |  | 184 (62.37%) | 139 (46.64 %) |  |  |
| Junior high school education | 1045 (26.02%) | 449 (25.95%) | 516 (30.48%) |  | 34 (11.53%) | 46 (15.44 %) |  |  |
| High school or technical secondary school education | 385 (9.59%) | 164 (9.48%) | 210 (12.40%) |  | 3 (1.02%) | 8 (2.68 %) |  |  |
| College education or above | 199 (4.96%) | 71 (4.10%) | 124 (7.32%) |  | 2 (0.68%) | 2 (0.67 %) |  |  |
| Careers, n(%) |  |  |  | 0.011 |  |  | 0.239 | <0.001 |
| Manual laborers | 3066 (76.35%) | 1330 (76.88%) | 1238 (73.12%) |  | 253 (85.76%) | 245 (82.21 %) |  |  |
| Intellectual workers | 950 (23.65%) | 400 (23.12%) | 455 (26.88%) |  | 42 (14.24%) | 53 (17.79 %) |  |  |
| HS, n(%) |  |  |  | <0.001 |  |  | 0.002 | 0.038 |
| Not self-sufficient | 201 (5.01%) | 133 (7.69%) | 49 (2.89%) |  | 16 (5.42%) | 3 (1.01 %) |  |  |
| Self-sufficient | 3815 (94.99%) | 1597 (92.31%) | 1644 (97.11%) |  | 279 (94.58%) | 295 (98.99 %) |  |  |
| HOED, n(%) |  |  |  | <0.001 |  |  | 0.902 | 0.001 |
| No | 3941 (98.13%) | 1669 (96.47%) | 1679 (99.17%) |  | 295 (100%) | 298 (100.00 %) |  |  |
| Yes | 75 (1.87%) | 61 (3.53%) | 14 (0.83%) |  | 0 (0%) | 0 (0.00 %) |  |  |
| HOG, n(%) |  |  |  | <0.001 |  |  | 0.012 | <0.001 |
| No | 2693 (67.06%) | 1085 (62.72%) | 1252 (73.95%) |  | 192 (65.08%) | 164 (55.03 %) |  |  |
| Yes | 1323 (32.94%) | 645 (37.28%) | 441 (26.05%) |  | 103 (34.92%) | 134 (44.97 %) |  |  |
| HGDU, n(%) |  |  |  | 0.138 |  |  | 0.187 | 0.009 |
| No | 3881 (96.64%) | 1670 (96.53%) | 1649 (97.40%) |  | 276 (93.56%) | 286 (95.97 %) |  |  |
| Yes | 135 (3.36%) | 60 (3.47%) | 44 (2.60%) |  | 19 (6.44%) | 12 (4.03 %) |  |  |
| HPI, n(%) |  |  |  | 0.376 |  |  | 0.497 | 0.044 |
| No | 3975 (98.98%) | 1707 (98.67%) | 1676 (98.99%) |  | 294 (99.66%) | 298 (100.00 %) |  |  |
| Yes | 41 (1.02%) | 23 (1.33%) | 17 (1.01%) |  | 1 (0.34%) | 0 (0.00 %) |  |  |
| HPV, n(%) |  |  |  | 0.621 |  |  | 0.015 | <0.001 |
| No | 4006 (99.75%) | 1729 (99.94%) | 1691 (99.88%) |  | 295 (100%) | 291 (97.65 %) |  |  |
| Yes | 10 (0.25%) | 1 (0.06%) | 2 (0.12%) |  | 0 (0%) | 7 (2.35 %) |  |  |
| HOOD, n(%) |  |  |  | 0.030 |  |  | 0.902 | <0.001 |
| No | 3862 (96.17%) | 1639 (94.74%) | 1630 (96.28%) |  | 295 (100%) | 298 (100.00 %) |  |  |
| Yes | 154 (3.83%) | 91 (5.26%) | 63 (3.72%) |  | 0 (0%) | 0 (0.00 %) |  |  |
| HOH, n(%) |  |  |  | 0.201 |  |  | 0.027 | <0.001 |
| No | 3528 (87.85%) | 1492 (86.24%) | 1485 (87.71%) |  | 281 (95.25%) | 270 (90.60 %) |  |  |
| Yes | 488 (12.15%) | 238 (13.76%) | 208 (12.29%) |  | 14 (4.75%) | 28 (9.40 %) |  |  |
| HOD, n(%) |  |  |  | 0.348 |  |  | 1.000 | <0.001 |
| No | 3844 (95.72%) | 1652 (95.49%) | 1605 (94.80%) |  | 292 (98.98%) | 295 (98.99 %) |  |  |
| Yes | 172 (4.28%) | 78 (4.51%) | 88 (5.20%) |  | 3 (1.02%) | 3 (1.01 %) |  |  |
| FHOEC, n(%) |  |  |  | 0.007 |  |  | 0.902 | <0.001 |
| No | 3799 (94.60%) | 1601 (92.54%) | 1605 (94.80%) |  | 295 (100%) | 298 (100.00 %) |  |  |
| Yes | 217 (5.40%) | 129 (7.46%) | 88 (5.20%) |  | 0 (0%) | 0 (0.00 %) |  |  |
| YOS, years,  M ± SD | 13.96 ± 19.51 | 16.08 ± 20.11 | 9.74 ± 17.77 | <0.001 | 25.93 ± 19.07 | 13.69 ± 19.07 | <0.001 | <0.001 |
| YOD, years,  M ± SD | 10.65 ± 17.75 | 13.58 ± 18.97 | 6.86 ± 15.47 | <0.001 | 17.99 ± 19.13 | 7.96 ± 15.73 | <0.001 | <0.001 |
| HOPE, n(%) |  |  |  | <0.001 |  |  | 0.523 | <0.001 |
| No | 3197 (79.61%) | 1232 (71.21%) | 1420 (83.87%) |  | 269 (91.19%) | 276 (92.62 %) |  |  |
| Yes | 819 (20.39%) | 498 (28.79%) | 273 (16.13%) |  | 26 (8.81%) | 22 (7.38 %) |  |  |
| LI, n(%) |  |  |  | 0.034 |  |  | 0.004 | <0.001 |
| None | 523 (13.02%) | 295 (17.05%) | 210 (12.40%) |  | 12 (4.07%) | 6 (2.01 %) |  |  |
| Light | 1800 (44.82%) | 727 (42.02%) | 876 (51.74%) |  | 112 (37.97%) | 85 (28.52 %) |  |  |
| Moderate | 1257 (31.30%) | 424 (24.51%) | 465 (27.47%) |  | 166 (56.27%) | 202 (67.79 %) |  |  |
| Heavy | 436 (10.86%) | 284 (16.42%) | 142 (8.39%) |  | 5 (1.69%) | 5 (1.68 %) |  |  |
| NS, n(%) |  |  |  | <0.001 |  |  | <0.001 | <0.001 |
| Poor | 404 (10.06%) | 343 (19.83%) | 52 (3.07%) |  | 8 (2.71%) | 1 (0.34 %) |  |  |
| Fair | 2264 (56.37%) | 1151 (66.53%) | 953 (56.29%) |  | 109 (36.95%) | 51 (17.11 %) |  |  |
| Good | 1348 (33.57%) | 236 (13.64%) | 688 (40.64%) |  | 178 (60.34%) | 246 (82.55 %) |  |  |
| OC, every day, n(%) |  |  |  | <0.001 |  |  | 0.056 | <0.001 |
| None | 432 (10.76%) | 249 (14.39%) | 155 (9.16%) |  | 10 (3.39%) | 18 (6.04 %) |  |  |
| 1-3 times | 3496 (87.05%) | 1457 (84.22%) | 1479 (87.36%) |  | 281 (95.25%) | 279 (93.62 %) |  |  |
| More then 3 times | 88 (2.19%) | 24 (1.39%) | 59 (3.48%) |  | 4 (1.36%) | 1 (0.34 %) |  |  |
| TODW, n(%) |  |  |  | <0.001 |  |  | 0.070 | <0.001 |
| Treated | 784 (19.52%) | 426 (24.62%) | 308 (18.19%) |  | 31 (10.51%) | 19 (6.38 %) |  |  |
| Untreated | 3232 (80.48%) | 1304 (75.38%) | 1385 (81.81%) |  | 264 (89.49%) | 279 (93.62 %) |  |  |
| SI, n(%) |  |  |  | <0.001 |  |  | <0.001 | 0.298 |
| Dislike | 1120 (27.89%) | 391 (22.60%) | 585 (34.55%) |  | 45 (15.25%) | 99 (33.22 %) |  |  |
| Medium | 1760 (43.82%) | 637 (36.82%) | 803 (47.43%) |  | 145 (49.15%) | 175 (58.72 %) |  |  |
| Like | 1136 (28.29%) | 702 (40.58%) | 305 (18.02%) |  | 105 (35.59%) | 24 (8.05 %) |  |  |
| PFHF, n(%) |  |  |  | <0.001 |  |  | <0.001 | <0.001 |
| No | 2051 (51.07%) | 754 (43.58%) | 1203 (71.06%) |  | 31 (10.51%) | 63 (21.14 %) |  |  |
| Yes | 1965 (48.93%) | 976 (56.42%) | 490 (28.94%) |  | 264 (89.49%) | 235 (78.86 %) |  |  |
| PFHFS, n(%) |  |  |  | <0.001 |  |  | <0.001 | <0.001 |
| No | 2558 (63.70%) | 969 (56.01%) | 1257 (74.25%) |  | 94 (31.86%) | 238 (79.87 %) |  |  |
| Yes | 1458 (36.30%) | 761 (43.99%) | 436 (25.75%) |  | 201 (68.14%) | 60 (20.13 %) |  |  |
| PFRF, n(%) |  |  |  | 0.359 |  |  | 0.902 | <0.001 |
| No | 3757 (93.55%) | 1592 (92.02%) | 1572 (92.85%) |  | 295 (100%) | 298 (100.00 %) |  |  |
| Yes | 259 (6.45%) | 138 (7.98%) | 121 (7.15%) |  | 0 (0%) | 0 (0.00 %) |  |  |
| PFPF, n(%) |  |  |  | <0.001 |  |  | <0.001 | 0.021 |
| None | 488 (12.15%) | 216 (12.49%) | 223 (13.17%) |  | 25 (8.47%) | 24 (8.05 %) |  |  |
| Dislike | 1220 (30.38%) | 466 (26.94%) | 544 (32.13%) |  | 72 (24.41%) | 138 (46.31 %) |  |  |
| Medium | 1328 (33.07%) | 452 (26.13%) | 627 (37.03%) |  | 142 (48.14%) | 107 (35.91 %) |  |  |
| Like | 980 (24.40%) | 596 (34.45%) | 299 (17.66%) |  | 56 (18.98%) | 29 (9.73 %) |  |  |
| PFSG, n(%) |  |  |  | <0.001 |  |  | 0.007 | <0.001 |
| No | 3800 (94.62%) | 1600 (92.49%) | 1614 (95.33%) |  | 288 (97.63%) | 298 (100.00 %) |  |  |
| Yes | 216 (5.38%) | 130 (7.51%) | 79 (4.67%) |  | 7 (2.37%) | 0 (0.00 %) |  |  |
| IOMF, n(%) |  |  |  | <0.001 |  |  | 0.030 | <0.001 |
| No | 3784 (94.22%) | 1565 (90.46%) | 1631 (96.34%) |  | 290 (98.31%) | 298 (100.00 %) |  |  |
| Yes | 232 (5.78%) | 165 (9.54%) | 62 (3.66%) |  | 5 (1.69%) | 0 (0.00 %) |  |  |
| PFSP, n(%) |  |  |  | 0.391 |  |  | 0.013 | 0.054 |
| Dislike | 1159 (28.86%) | 498 (28.79%) | 510 (30.12%) |  | 62 (21.02%) | 89 (29.87 %) |  |  |
| Like | 2857 (71.14%) | 1232 (71.21%) | 1183 (69.88%) |  | 233 (78.98%) | 209 (70.13 %) |  |  |
| ROE, n(%) |  |  |  | <0.001 |  |  | <0.001 | <0.001 |
| Slow | 727 (18.10%) | 286 (16.53%) | 339 (20.02%) |  | 36 (12.20%) | 66 (22.15 %) |  |  |
| Medium | 1937 (48.23%) | 691 (39.94%) | 1011 (59.72%) |  | 57 (19.32%) | 178 (59.73 %) |  |  |
| Quick | 1352 (33.67%) | 753 (43.53%) | 343 (20.26%) |  | 202 (68.47%) | 54 (18.12 %) |  |  |
| EADU, n(%) |  |  |  | <0.001 |  |  | 0.009 | 0.158 |
| No | 3631 (90.41%) | 1468 (84.86%) | 1617 (95.51%) |  | 263 (89.15%) | 283 (94.97 %) |  |  |
| Yes | 385 (9.59%) | 262 (15.14%) | 76 (4.49%) |  | 32 (10.85%) | 15 (5.03 %) |  |  |
| VI, n(%) |  |  |  | <0.001 |  |  | <0.001 | <0.001 |
| None | 19 (0.47%) | 12 (0.69%) | 5 (0.30%) |  | 2 (0.68%) | 0 (0.00 %) |  |  |
| Dislike | 353 (8.79%) | 189 (10.92%) | 82 (4.84%) |  | 69 (23.39%) | 13 (4.36 %) |  |  |
| Medium | 1552 (38.65%) | 705 (40.75%) | 592 (34.97%) |  | 124 (42.03%) | 131 (43.96 %) |  |  |
| Like | 2092 (52.09%) | 824 (47.63%) | 1014 (59.89%) |  | 100 (33.90%) | 154 (51.68 %) |  |  |
| FI, n(%) |  |  |  | <0.001 |  |  | <0.001 | 0.002 |
| None | 181 (4.51%) | 134 (7.75%) | 41 (2.42%) |  | 6 (2.03%) | 0 (0.00 %) |  |  |
| Dislike | 1157 (28.81%) | 602 (34.80%) | 399 (23.57%) |  | 119 (40.34%) | 37 (12.42 %) |  |  |
| Medium | 1558 (38.80%) | 620 (35.84%) | 684 (40.40%) |  | 109 (36.95%) | 145 (48.66 %) |  |  |
| Like | 1120 (27.88%) | 374 (21.62%) | 569 (33.61%) |  | 61 (20.68%) | 116 (38.93 %) |  |  |
| TI, n(%) |  |  |  | <0.001 |  |  | <0.001 | <0.001 |
| None | 1553 (38.67%) | 707 (40.87%) | 781 (46.13%) |  | 31 (10.51%) | 36 (12.08 %) |  |  |
| Dislike | 1558 (38.80%) | 649 (37.51%) | 681 (40.22%) |  | 67 (22.71%) | 161 (54.03 %) |  |  |
| Like | 903 (22.49%) | 374 (21.62%) | 231 (13.64%) |  | 197 (66.78%) | 101 (33.89 %) |  |  |
| GI, n(%) |  |  |  | 0.098 |  |  | 0.003 | <0.001 |
| Less | 1140 (28.39%) | 559 (32.31%) | 540 (31.90%) |  | 19 (6.44%) | 22 (7.38 %) |  |  |
| Medium | 2133 (53.11%) | 835 (48.27%) | 907 (53.57%) |  | 178 (60.34%) | 213 (71.48 %) |  |  |
| More | 743 (18.50%) | 336 (19.42%) | 246 (14.53%) |  | 98 (33.22%) | 63 (21.14 %) |  |  |
| CI, n(%) |  |  |  | <0.001 |  |  | 0.041 | <0.001 |
| Less | 1513 (37.67%) | 702 (40.58%) | 764 (45.13%) |  | 35 (11.86%) | 12 (4.03 %) |  |  |
| Medium | 1727 (43.00%) | 703 (40.64%) | 716 (42.29%) |  | 148 (50.17%) | 160 (53.69 %) |  |  |
| More | 776 (19.32%) | 325 (18.79%) | 213 (12.58%) |  | 112 (37.97%) | 126 (42.28 %) |  |  |
| COI, every day, n(%) |  |  |  | 0.045 |  |  | 0.584 | <0.001 |
| Less than 25g | 1048 (26.10%) | 533 (30.81%) | 468 (27.64%) |  | 23 (7.80%) | 24 (8.05 %) |  |  |
| 25-50g | 2333 (58.09%) | 904 (52.25%) | 915 (54.05%) |  | 254 (86.10%) | 260 (87.25 %) |  |  |
| More then 50g | 635 (15.81%) | 293 (16.94%) | 340 (20.08%) |  | 18 (6.10%) | 14 (4.70 %) |  |  |
| MI, every day, n(%) |  |  |  | 0.021 |  |  | 0.427 | <0.001 |
| Less than 250g | 1284 (31.97%) | 658 (38.03%) | 590 (34.85%) |  | 16 (5.42%) | 20 (6.71 %) |  |  |
| 250-500g | 1820 (45.32%) | 766 (44.28%) | 760 (44.89%) |  | 144 (48.81%) | 150 (50.34 %) |  |  |
| More then 500g | 912 (22.71%) | 306 (17.69%) | 343 (20.26%) |  | 135 (45.76%) | 128 (42.95 %) |  |  |

*All variables were based on the questionnaire items and met any of the following criteria: 1) baseline demographic information; 2) biological plausibility for predicting EC risk; 3) reported in published studies on EC risk factors.Categorical variables are expressed as frequency and percentage (n, %), Continuous variables are expressed as mean ± standard deviation (M ± SD).

Specific information about certain variables：

1) Marital status is divided into unmarried and married (including separated, divorced, and widowed). 2) Geographical characteristic is divided into more livable (mainly referring to plains and regions with suitable climates) and less livable (mainly referring to mountainous regions and regions with dry or humid climates). 3) Labor intensity is categorized into four levels: none (no physical labor or minimal physical exertion), light (minimal physical exertion, such as office clerks or typists), moderate (moderate physical exertion with intermittent rest periods, such as teachers or sales clerks), and heavy (significant physical exertion requiring prolonged labor, such as porters or construction workers). 4) Nutritional status is categorized as poor (BMI < 18.5, with low dietary intake, low muscle mass, and poor physical fitness), fair (18.5 ≤ BMI, with unbalanced dietary intake, moderate muscle mass, and average physical fitness), and good (18.5 ≤ BMI, with balanced dietary intake, adequate muscle mass, and good physical fitness) (Body Mass Index (BMI) = weight (kg) / height (m)²). 5) Dietary habit variables are categorized based on actual conditions, such as SI, which is divided into dislike (unpleasant but necessary to consume), medium (acceptable), and like (prefers to consume salty foods). Preference for hot food is divided into no (prefers cold food over hot food) and yes (prefers hot food over cold food). Preference for pickled food is categorized as none (does not consume pickled foods), dislike (does not like but consumes in small amounts), medium (acceptable), and like (likes and frequently consumes). Consuming less then 3 times per week is considered small intake, 3–5 times is moderate, and more than 5 times is frequent consumption.

*The P-value refers to the comparison between EC participants and non-EC participants in the training cohort.

#The P-value refers to the comparison between EC participants and non-EC participants in the external validation cohort.

The P-value refers to the comparison between the training cohort and the external validation cohort.
