# Supplementary material for: Development and external validation of a nomogram for predicting the risk of developing esophageal cancer based on a questionnaire: a multicenter case-control study
Source: Front Oncol. 2025 Dec 4;15:1684561. doi: 10.3389/fonc.2025.1684561 (PMC12711463; doi:10.3389/fonc.2025.1684561)
Supplement: Supplementary file 2 [file DataSheet1.docx]

Final Logistic Regression Model Equation:

logit(p) = α + β1*Sex + β2*NS + β3*Age + β4*PFPF + β5*PFHF + β6*PFHFS + β7*ROE + β8*VI + β9*FI + β10*COI

Specific coefficient values：

| Variable | Coefficient | | | | | | | |
| --- | --- | --- | --- | --- | --- | --- | --- | --- |
| Intercept | 0.728 | | | | | | | |
| Sex | Female | | | | Male | | | |
|  | 0 | | | | 0.566 | | | |
| NS | Poor | | | Fair | | Good | | |
|  | 0 | | | -1.548 | | -2.891 | | |
| Age | 0.016 | | | | | | | |
| PFPF | None | | Dislike | | Medium | | | Like |
|  | 0 | | 0.030 | | -0.339 | | | 0.466 |
| PFHF | No | | | | Yes | | | |
|  | 0 | | | | 0.871 | | | |
| PFHFS | No | | | | Yes | | | |
|  | 0 | | | | 0.241 | | | |
| ROE | Slow | | | Medium | | Quick | | |
|  | 0 | | | -0.283 | | 0.795 | | |
| VI | None | | Dislike | | Medium | | | Like |
|  | 0 | | 0.526 | | 0.345 | | | -0.108 |
| FI | None | Dislike | | | Medium | | Like | |
|  | 0 | -0.609 | | | -0.750 | | -0.805 | |
| COI | Less | | | Medium | | More | | |
|  | 0 | | | -0.422 | | -0.871 | | |

The nomogram 2 constructed based on 5 core predictors, along with its internal validation, external validation, and related information, is as follows:

logit(p) = α + β1*Sex + β2*NS + β3*PFPF + β4*ROE + β5*COI

| Variable | Coefficient | | | |
| --- | --- | --- | --- | --- |
| Intercept | 1.211 | | | |
| Sex | Female | | Male | |
|  | 0 | | 0.636 | |
| NS | Poor | Fair | | Good |
|  | 0 | -1.623 | | -3.026 |
| PFHF | No | | Yes | |
|  | 0 | | 1.141 | |
| ROE | Slow | Medium | | Quick |
|  | 0 | -0.303 | | 0.808 |
| COI | Less | Medium | | More |
|  | 0 | -0.393 | | -0.836 |


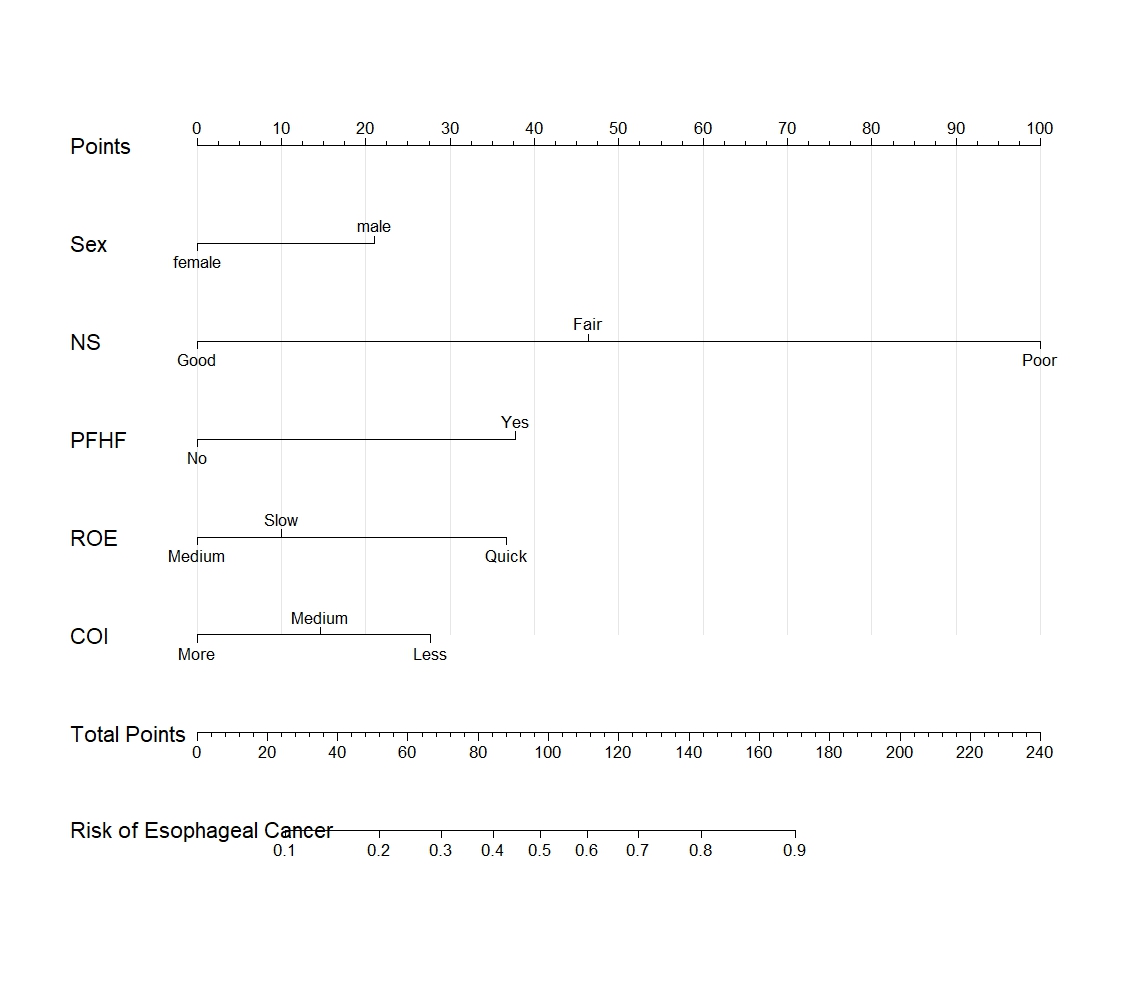


Nomogram 2 (based on 5 core predictors)


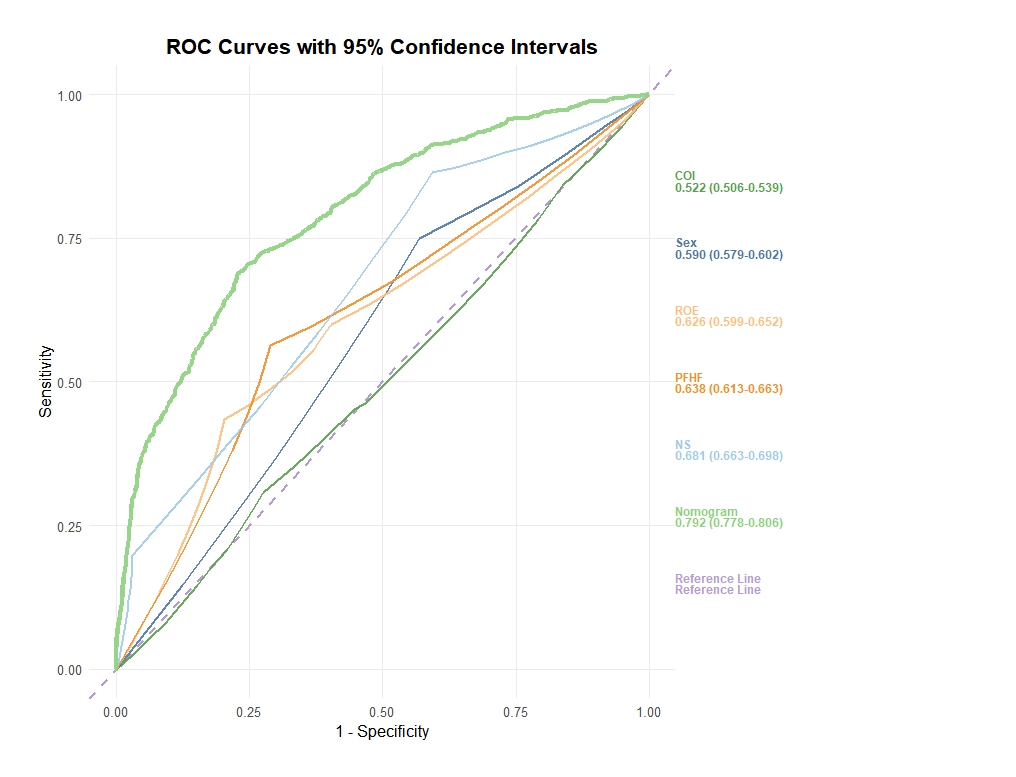


ROC curve of the nomogram 2 and single independent predictor in the training cohort.


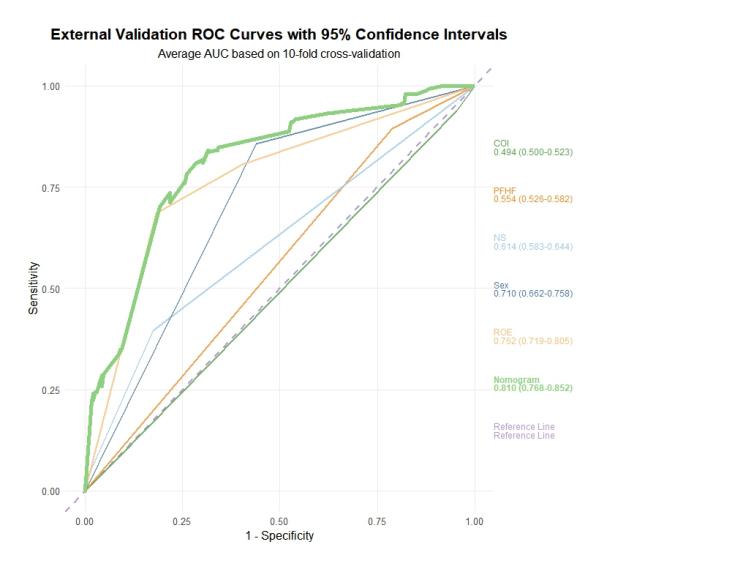


ROC curve of the nomogram 2 and single independent predictor in the external validation cohort.

|  | Internal validation: nomogram performance | | | External validation: nomogram performance | | |
| --- | --- | --- | --- | --- | --- | --- |
| Measure | Original value | Optimism | Corrected | Original value | Optimism | Corrected |
| AUC | 0.795 | 0.005 | 0.790 | 0.807 | 0.001 | 0.806 |
| *R*^2^ | 0.335 | 0.011 | 0.324 | 0.363 | -0.002 | 0.364 |
| Brier score | 0.185 | -0.002 | 0.187 | 0.178 | 0.000 | 0.178 |
| Slope | 1 | 0.025 | 0.975 | 1.244 | 0.014 | 1.230 |

Internal and external validation of nomogram 2 by the bootstrap method.


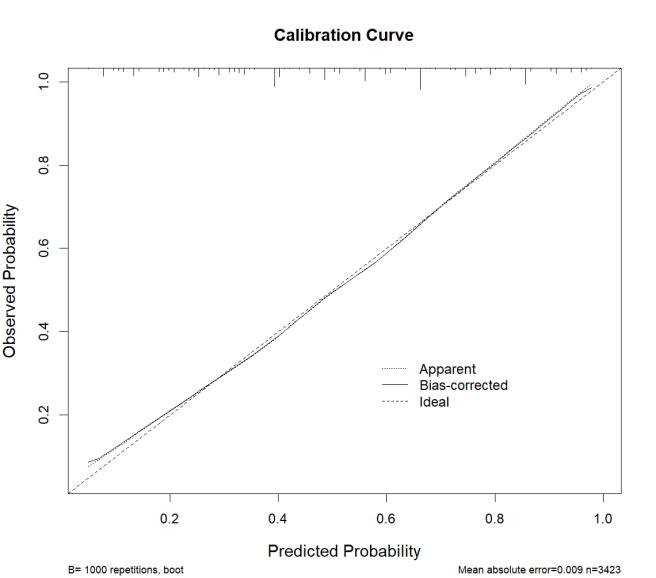


The calibration curve of the nomogram 2 in the training cohort.


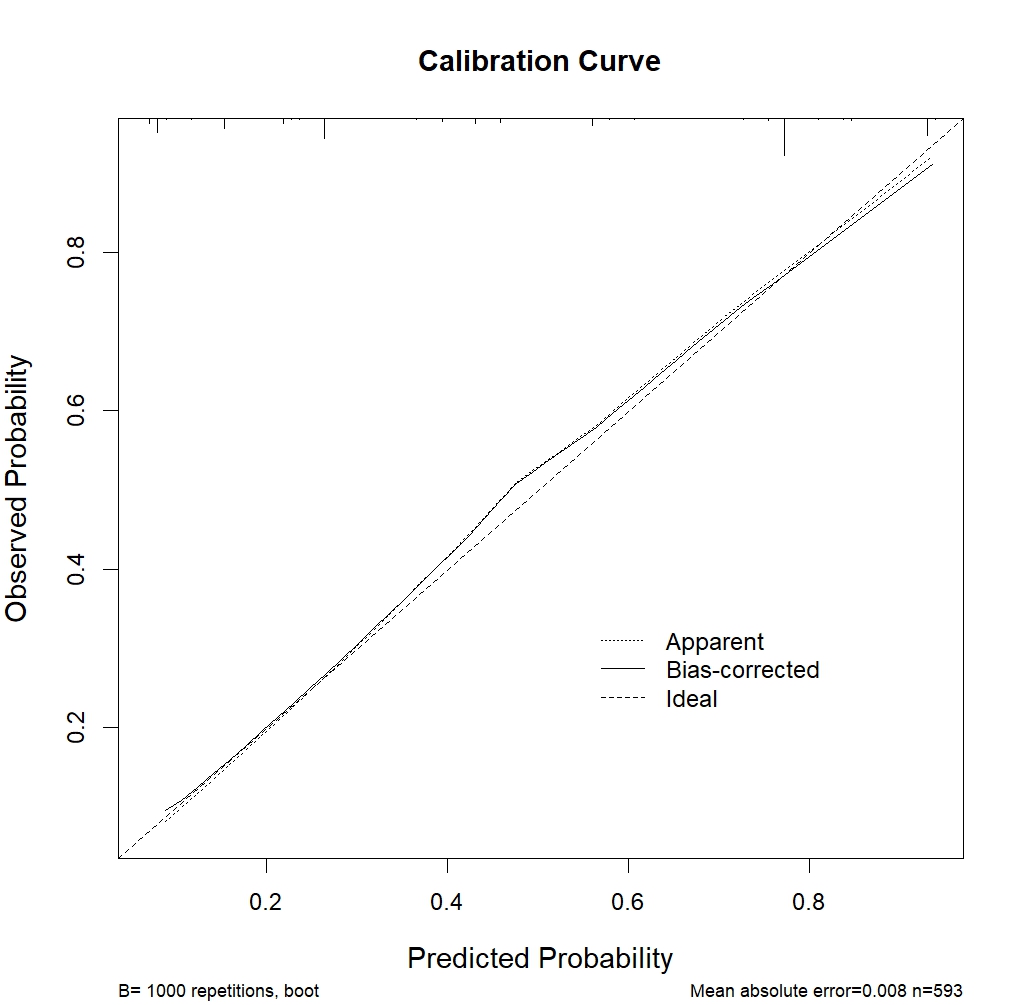


The calibration curve of the nomogram 2 in the external validation cohort.


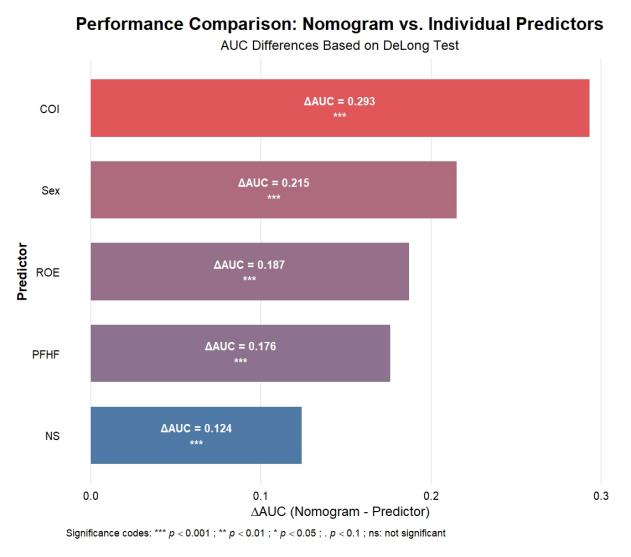


Delong test between nomogram 2 model and single independent predictor in the training cohort.


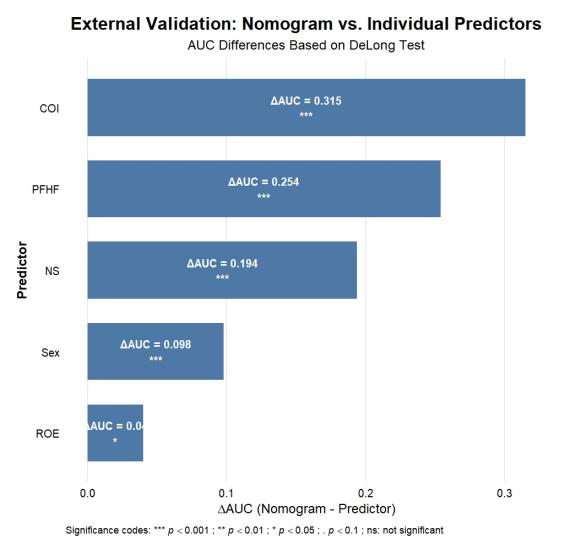


Delong test between nomogram 2 model and single independent predictor in the external validation cohort.


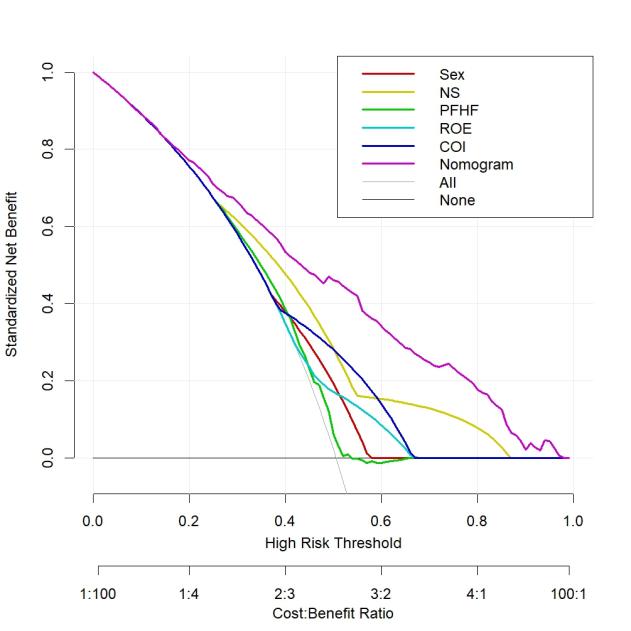


The decision curve analysis (DCA) of the nomogram 2 prediction in the training cohort.


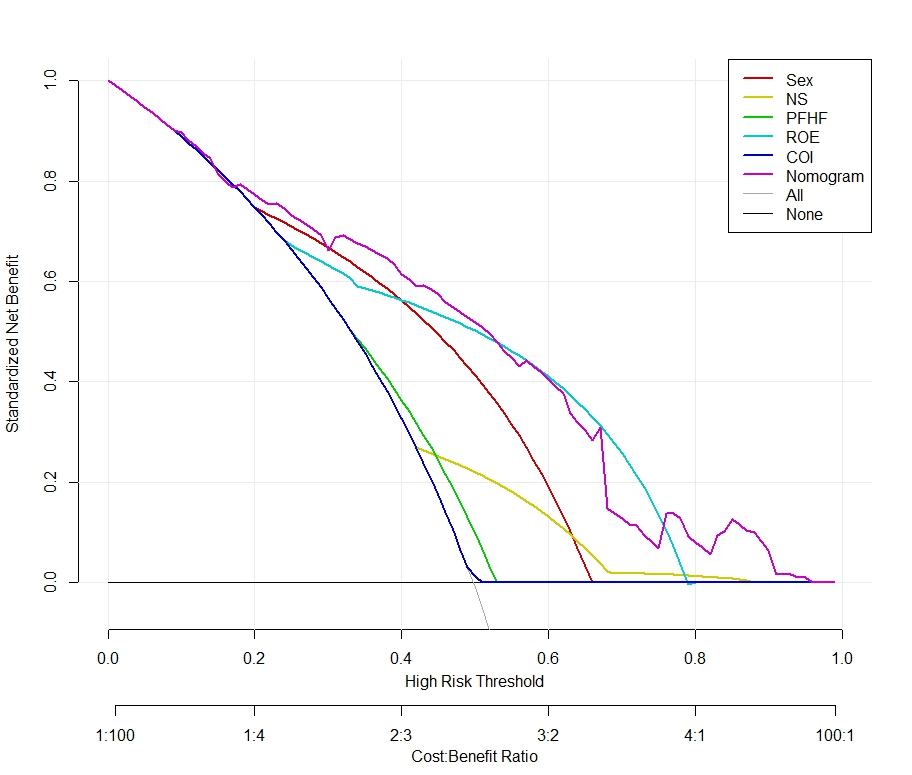


The decision curve analysis (DCA) of the nomogram 2 prediction in the external validation cohort.


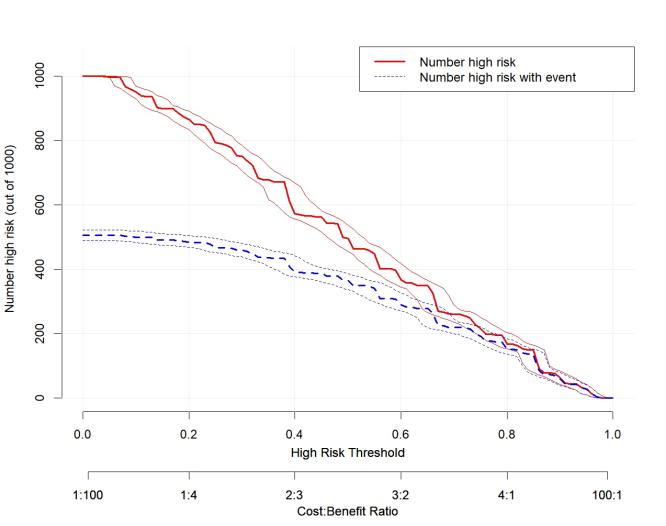


The clinical impact curve (CIC) of the nomogram prediction in the training cohort.


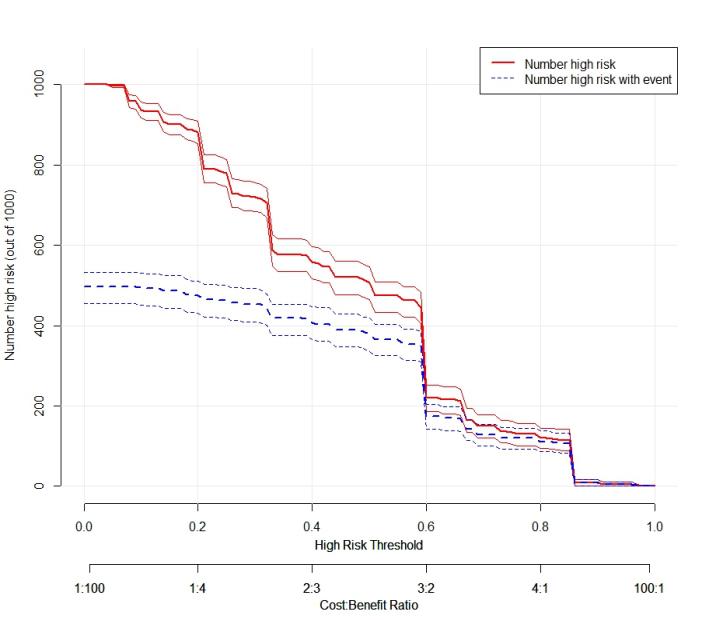


The clinical impact curve (CIC) of the nomogram prediction in the external validation cohort.
