# Supplementary material for: Development and external validation of a nomogram for predicting the risk of developing esophageal cancer based on a questionnaire: a multicenter case-control study
Source: Front Oncol. 2025 Dec 4;15:1684561. doi: 10.3389/fonc.2025.1684561 (PMC12711463; doi:10.3389/fonc.2025.1684561)
Supplement: Supplementary file 3 [file DataSheet2.docx]

We selected these five “clinically important” variables primarily based on three criteria: significant clinical relevance (literature demonstrating strong association with esophageal cancer incidence), clinical accessibility, and ease of quantification, with priority given to variables related to dietary habits.

1. Variables showing no significant association with esophageal cancer incidence were first excluded, including: PFSP, Careers, HOG, and HOPE. While HOPE exhibits some correlation with esophageal cancer, stringent national regulations in recent years have prohibited highly toxic pesticides, with the vast majority now being low-toxicity formulations. Consequently, this correlation has significantly diminished.

2. Variables with insufficient positive cases were then excluded, including HOED (75/3423). Although HOED shows a significant association with esophageal cancer incidence, its low positive case count limits its predictive contribution.

3. Finally, variables where accurate clinical information is difficult to obtain or quantify were excluded, including LI, SI, YOD, and EADU.

Regarding YOD and YOS, while studies indicate alcohol consumption and smoking are major causes of esophageal cancer, other research suggests these factors play a more significant role in adenocarcinoma development. and over 90% of esophageal cancers in China are squamous cell carcinomas. Most critically, unlike variables such as age and dietary habits, we cannot accurately quantify smoking and drinking levels. For example, two patients may both have smoked for 10 years, but their daily consumption could differ greatly; using “10 years” as a single metric for both is unreasonable. Dietary habits, however, are typically long-term behaviors.

Furthermore, while TI shows some correlation with esophageal cancer incidence, it exhibits significant overlap with PFHF. Therefore, we opted for the broader PFHF.

| Variables | Standard |
| --- | --- |
| Sex | Core Predictors |
| LI | difficult to quantify accurately |
| Age | Significant clinical significance, clinical accessibility, and ease of quantification^[71, 72]^ |
| HOED | Too few positive cases |
| HOG | No significant correlation |
| NS | Core Predictors |
| YOD | difficult to quantify accurately |
| SI | difficult to quantify accurately |
| PFHF | Core Predictors |
| PFSP | No significant correlation |
| PFPF | Significant clinical significance, clinical accessibility, and ease of quantification^[55-58]^ |
| ROE | Core Predictors |
| EADU | difficult to quantify accurately |
| VI | Significant clinical significance, clinical accessibility, and ease of quantification^[64-66]^ |
| FI | Significant clinical significance, clinical accessibility, and ease of quantification^[64-66]^ |
| TI | Overlap with PFHF (Preference for Hot Foods) |
| COI | Core Predictors |
| HOPE | No significant correlation |
| Careers | No significant correlation |
| PFHFS | Significant clinical significance, clinical accessibility, and ease of quantification^[37, 52]^ |
